# The Composition of Human Milk and Infant Faecal Microbiota Over the First Three Months of Life: A Pilot Study

**DOI:** 10.1038/srep40597

**Published:** 2017-01-17

**Authors:** Kiera Murphy, David Curley, Tom F. O’Callaghan, Carol-Anne O’Shea, Eugene M. Dempsey, Paul W. O’Toole, R. Paul Ross, C. Anthony Ryan, Catherine Stanton

**Affiliations:** 1 Food Biosciences, Teagasc Food Research Centre, Fermoy, Co Cork, Ireland; 2 School of Microbiology, University College Cork, Cork, Ireland; 3 APC Microbiome Institute, University College Cork, Cork, Ireland; 4 Department of Neonatology, Cork University Maternity Hospital, Cork, Ireland; 5 College of Science, Engineering and Food Science, University College Cork, Cork, Ireland

## Abstract

Human milk contains a diverse array of bioactives and is also a source of bacteria for the developing infant gut. The aim of this study was to characterize the bacterial communities in human milk and infant faeces over the first 3 months of life, in 10 mother-infant pairs. The presence of viable *Bifidobacterium* and *Lactobacillus* in human milk was also evaluated. MiSeq sequencing revealed a large diversity of the human milk microbiota, identifying over 207 bacterial genera in milk samples. The phyla Proteobacteria and Firmicutes and the genera *Pseudomonas, Staphylococcus* and *Streptococcus* were the predominant bacterial groups. A core of 12 genera represented 81% of the microbiota relative abundance in milk samples at week 1, 3 and 6, decreasing to 73% at week 12. Genera shared between infant faeces and human milk samples accounted for 70–88% of the total relative abundance in infant faecal samples, supporting the hypothesis of vertical transfer of bacteria from milk to the infant gut. In addition, identical strains of *Bifidobacterium breve* and *Lactobacillus plantarum* were isolated from the milk and faeces of one mother-infant pair. Vertical transfer of bacteria via breastfeeding may contribute to the initial establishment of the microbiota in the developing infant intestine.

Early microbial colonisation of the neonatal gut exerts a major effect on host health status^1^,^2^. Early colonization is influenced by a number of factors including feeding practice, with the intestinal microbiome composition differing in breast-fed and formula-fed infants^3^. Breast-fed infants have been reported to have a more stable bacterial population, while the microbiota of formula-fed infants appears to be more diverse, containing higher numbers of *E. coli, Clostridium difficile, Enterococcus, Enterobacter* and *Citrobacter*^4^,^5^,^6^. Formula-fed infants have been reported to have higher morbidity and mortality during the first year of life and have an increased risk of developing respiratory and gastrointestinal infections and diseases such as necrotising enterocolitis and sepsis, compared with breast-fed infants^7^,^8^.

The health promoting effects of human milk have been linked to the abundance of bioactive molecules present therein, including secretory antibodies, immune cells, antimicrobial proteins such as lactoferrin, CD14 and lysozyme, regulatory cytokines and human milk oligosaccharides (HMOs)^9^. Though indigestible by the infant, these HMOs exert immunomodulatory effects, stimulate the growth of commensal bacteria, particularly bifidobacteria and are involved in defence against pathogens by preventing adhesion to the gut epithelium during a vulnerable period in life, when the infant’s own defences are immature^10^.

It has been reported that human milk is in itself a source of commensal bacteria that may colonise the infant gut. Predominant culturable bacterial populations have been identified as *Staphylococcus* and *Streptococcus* species, while lactic acid bacteria and bifidobacteria have also been isolated^11^,^12^,^13^. In an earlier culture-independent study, *Staphylococcus* and *Streptococcus* dominated the milk microbiota of most mothers, while a more recent study revealed a larger microbial diversity of human milk than originally thought, with more than 700 species identified^14^,^15^. Interestingly, the study also reported that the human milk microbiota was compositionally distinct from other body niches and maternal weight and mode of delivery were reported as determinants of composition.

The origin of the human milk microbiome has been the subject of debate. Possible origins include bacteria from maternal skin and the oral cavity of the infant, where it has been demonstrated that during suckling, a high degree of retrograde flow back into the mammary ducts occurs^16^. More recently, the maternal gut has been suggested as a source, with bacteria entering the mammary glands via the entero-mammary pathway, a route that involves phagocytic dendritic cells penetrating the gut epithelium and trafficking bacteria through the circulatory system^17^,^18^,^19^. This is important since manipulation of the maternal microbiota could be used to promote an optimal human milk microbiome.

Given the significant health effects that breast-feeding exerts on infant health and development, it is important to define the composition of the human milk microbiota. Accordingly, the aim of this study was to characterise the microbiota composition of human milk in ten lactating women, and correlate the findings with gut microbiota composition of their infants, over the first three months of life. Additionally, we assessed the presence of culturable *Bifidobacterium* and *Lactobacillus* strains and their recovery from the infant gut.

## Results

### MiSeq Sequencing of Human Milk and Infant Faeces

MiSeq sequencing of human milk and infant faecal samples yielded a total of 20,206,055 reads, with mean read lengths of 460 bp (330–591 bp). Following quality control, an average of 61,786 (range 34836–109067) and 189,861 (range 98772–327481) reads per sample were obtained for human milk and infant faeces, respectively. Reads were classified into 1313 observed operational taxonomic units (OTUs) for human milk and 264 OTUs for infant faeces at a 3% similarity cut-off. All samples were rarefied to 33,000 sequences to prevent bias due to sampling depth.

**Figure 1:**
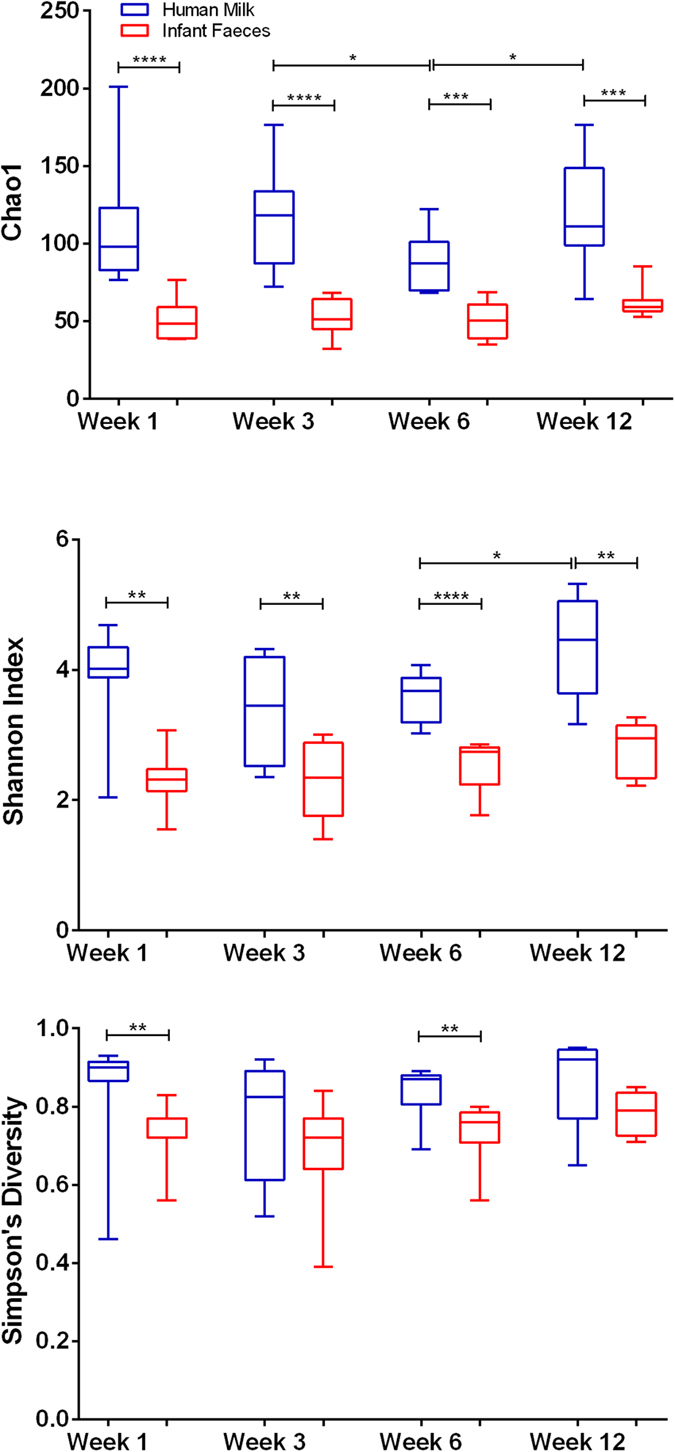
Alpha diversity estimates (**a**) Chao1 (**b**) Shannon index (**c**) Simpson’s diversity index, for human milk and infant faeces samples over time. ****Significant at p ≤ 0.0001; ***significant at p ≤ 0.001; **significant at p ≤ 0.01; *significant at p ≤ 0.05.

To estimate microbial richness we used the Chao1 richness estimator which revealed significantly higher bacterial richness in milk samples than faecal samples at week 1, 3, 6 and 12 (p < 0.0001, p < 0.0001, p = 0.0002 and p = 0.0003 respectively) (Fig. 1). We applied the Simpson’s index to estimate microbial evenness and this was significantly higher in milk samples at week 1 and 6 (p = 0.002 and p = 0.004). To predict microbial diversity we used the Shannon index, which combines both bacterial richness and evenness, and is more responsive to rare species in terms of species richness than the Simpson’s index which is more sensitive to dominant species. The Shannon index was significantly higher in milk samples at week 1, 3, 6 and 12 (p = 0.0012, p = 0.005, p < 0.0001 and p = 0.004). Over time, there was a significant increase in bacterial richness, Chao1, from week 3 to 6 (p = 0.0462) and a significant decrease in richness from week 6 to 12 (p = 0.0462) in milk samples. The Shannon index demonstrated a significant decrease in diversity in milk samples from week 6 to 12 (p = 0.0482). There were no statistically significant differences in alpha diversity metrics in fecal samples over time.

**Figure 2:**
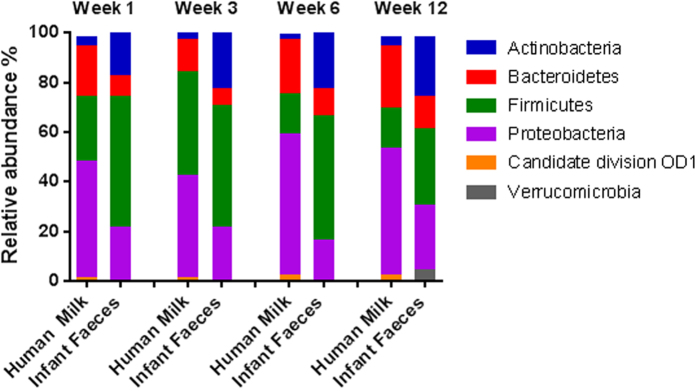
Phylum level assignments of average relative abundances of the microbiota in human milk and infant faeces over week 1, 3, 6 and 12.

Regarding phyla, at week 1, human milk was dominated by Proteobacteria (41%), Firmicutes (35%) and Bacteroidetes (17%), accounting for 93% of all reads (Fig. 2). Conversely, infant faeces harboured higher relative abundances of Firmicutes (56%) and Actinobacteria (20%) and lower Proteobacteria (21%) and Bacteroidetes (3%). Proteobacteria dominated in milk samples, with the exception of week 3 where Firmicutes showed the highest mean relative abundance (50%).

**Figure 3:**
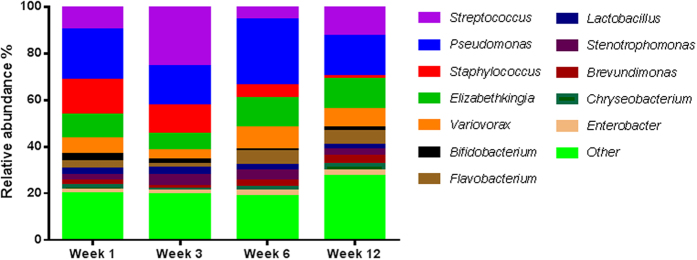
The core genera and average relative abundances identified in the microbiota of human milk. Core as defined by the presence in the microbiota of 90% or more of the 10 women at ≥1% of the total reads.

At genus level, 12 genera appeared to predominate in human milk, as they were detected at a mean relative abundance of ≥1% in at least 90% of samples collected over time (Fig. 3). These genera dominated the community, representing 81% of the relative abundance in human milk samples at week 1 (range 64–91%), 3 (range 64–90%) and 6 (range 61–90%) and 73% of the relative abundance at week 12 (range 50–82%). This core comprised *Pseudomonas, Staphylococcus, Streptococcus, Elizabethkingia, Variovorax, Bifidobacterium, Flavobacterium, Lactobacillus, Stenotrophomonas, Brevundimonas, Chryseobacterium* and *Enterobacter*. The remaining sequences mapped to 195 genera, further demonstrating the diversity of the human milk microbiota (Supplementary Table S1). The relative abundances of these genera were individual specific and subject to intra-individual variations over time.

**Figure 4:**
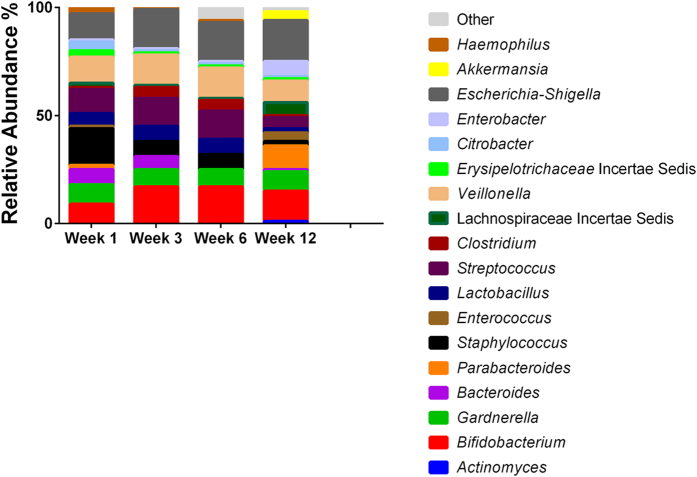
Genus level assignments of average relative abundances of the faecal microbiota in infants.

There was little temporal stability in milk samples from most lactating women, as the relative abundance of the bacterial genera present shifted over time (Supplementary Table S1). With the exception of week 3 where *Streptococcus* had the highest mean relative abundance (32%), *Pseudomonas* was predominant at all other sampling points detected at 21%, 27% and 19% at week 1, 6 and 12 respectively. By contrast, only 50 genera were detected in infant faecal samples. *Pseudomonas* was not detected by sequencing and the samples were dominated by *Staphylococcus* at week 1 (19%), *Escherichia-Shigella* at week 3 and 12 (17% and 21% respectively) and *Veillonella* at week 6 (23%) (Fig. 4). When considering genera detected in both human milk and faeces (Table 1), these accounted for 88%, 85%, 88% and 70% of the total reads in infant faecal samples at week 1, 3, 6 and 12 respectively. For human milk, these genera accounted for 37%, 51%, 19% and 27% of the total reads. These genera occurred with varying relative abundances and frequencies. *Veillonella* and *Escherichia/Shigella* for example, occurred at a higher mean relative abundance in infant faeces, 14% and 12% respectively, compared to 1% and 0.04% respectively in human milk at week 1. In contrast, *Staphylococcus* was consistently detected at a high frequency in all samples with a mean relative abundance of 13% and 19% at week 1 in human milk and infant faeces, respectively.

A number of niche-specific genera were exclusive to either human milk or infant faeces. For example, the high relative abundance of Proteobacteria and Bacteroidetes in human milk was largely attributable to the genera *Pseudomonas* and *Variovorax*, and *Elizabethkingia* and *Flavobacterium* respectively, which were exclusive to human milk. In infant faeces, *Eggerthella*, which was the only genus not detected in human milk samples, contributed to the higher relative abundance of Actinobacteria.

One subject, M10, reported symptoms of subacute mastitis and withdrew from the study at week 6 following antibiotic administration. Among the milk samples obtained from this subject, the relative abundance of *Staphylococcus* was higher than the mean relative abundance for healthy subjects, accounting for 73% and 24% of the reads obtained at week 1 and week 3, respectively, compared to 12% and 6% in healthy women. Alpha diversity metrics were also lowest in the mastitic milk, this likely reflects the low abundance of other species in this sample. Also of note was mother-infant pair 9, where *Haemophilus* was detected at a relative abundance of 24% in the human milk sample and 33% in the infant fecal microbiota at week 1. The mean relative abundance for *Haemophilus* in other human milk samples at week 1 was 0.1% and 0.5% in infant faeces.

Principal coordinate analysis (PCoA) plots using weighted UniFrac distances demonstrated a clear separation of milk and faecal samples at each week (Supplementary Figure 1). There were no distinct clusters of milk samples over time, although bacterial communities clustered more closely to one another at week 12 than at week 1 (Supplementary Figure 2a). Similarly, in faecal samples no obvious separation was observed between samples at different weeks (Supplementary Figure 2b).

### Culture-Dependent Analysis of Human Milk and Infant Faeces

**Figure 5:**
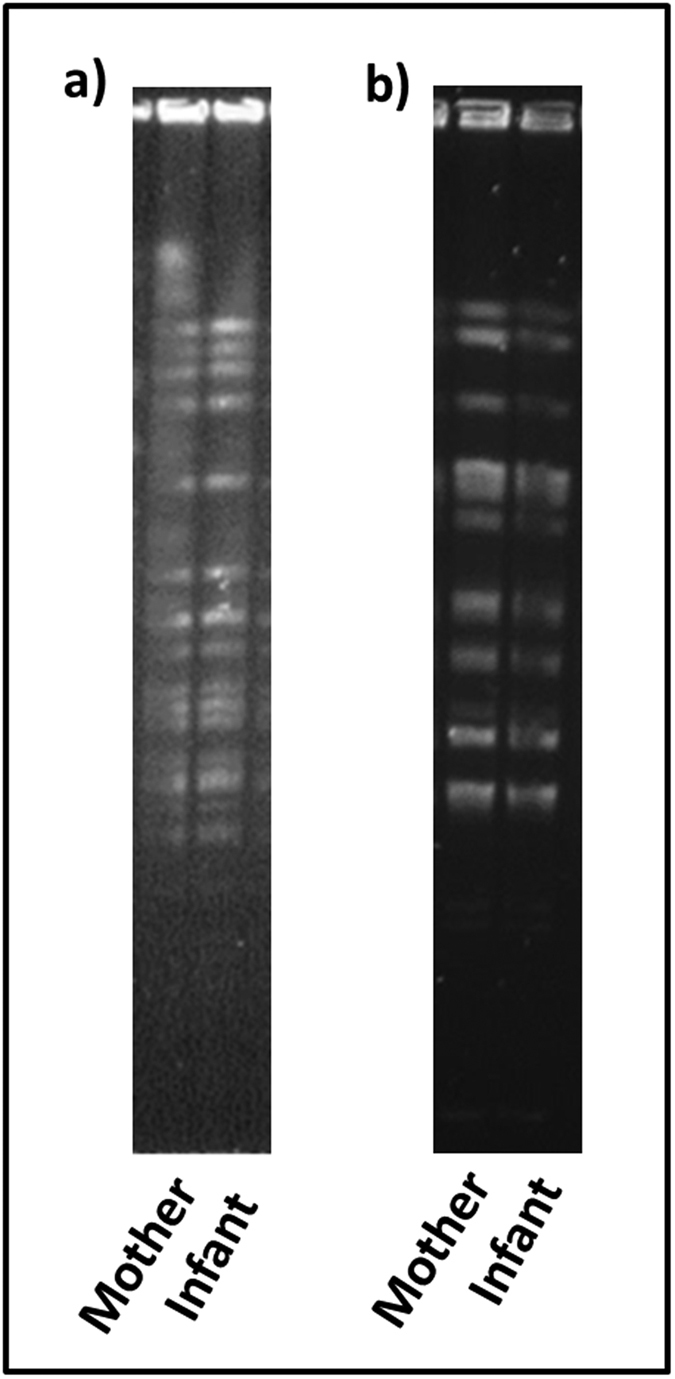
Pulse-field gel electrophoresis patterns of (**a**) XbaI-digested genomic DNA of *B. breve* isolates and (**b**) ApaI-digested genomic DNA of *L. plantarum* isolates from human milk and infant faeces. The unedited versions of these images can be found as Supplementary Figures S3 and S4.

Culture on selective media revealed the presence of presumptive *Bifidobacterium* and *Lactobacillus* in the human milk of one mother at 1 × 10^2^ CFU/ml and 3 × 10^3^ CFU/ml respectively at week 3. *Bifidobacterium* and *Lactobacillus* were isolated from the corresponding infant fecal sample at 4 × 10^6^ CFU/ml and 7 × 10^7^ CFU/ml, respectively. No culturable *Bifidobacterium* or *Lactobacillus* were detected in human milk samples from other mothers in the study. Following enumeration, bacterial isolates from the human milk and the corresponding infant stool sample were subjected to 16S rRNA gene sequencing using species-specific primers. This identified the *Bifidobacterium* isolates as *B. breve* and *Lactobacillus* isolates as *L. plantarum* in both the human milk and the infants fecal samples. In addition, PFGE analysis of these isolates revealed identical profiles in both the human milk and the infant fecal samples for both *B. breve* and *L. plantarum,* which indicated that these isolates belonged to the same strain (Fig. 5).

## Discussion

The benefits of human milk in terms of infant health and development have been well documented^1^,^8^,^10^,^12^,^17^,^20^. Human milk has been recognized as a fundamental source of bioactive components including bacteria that may contribute to neonatal gastrointestinal colonisation and immune development and maturation during the crucial early stages of development^8^,^9^,^10^. Differences have been reported in the microbiota composition of breast-fed infants versus formula-fed with the former suffering from less allergies and gastrointestinal infections^3^,^4^. Therefore, the microbiota of breast-fed infants is considered the gold standard in terms of a healthy infant gastrointestinal microbiota. Comprehensively characterising the human milk microbiota is vital for enabling better insight of its significance and activity in relation to the developing infant gut microbiota and health.

In this study, Illumina MiSeq sequencing revealed that 12 genera; *Pseudomonas, Staphylococcus, Streptococcus, Elizabethkingia, Variovorax, Bifidobacterium, Flavobacterium, Lactobacillus, Stenotrophomonas, Brevundimonas, Chryseobacterium* and *Enterobacter* dominated the milk of most lactating women, constituting a core milk microbiota. The presence and relative abundances of the remaining 195 genera were unique to individual mothers and subject to variation over time. The core microbiota constituted 81% of the taxa identified at week 1, 3 and 6, however by week 12, this decreased to 73%, suggesting a selected core microbiota drives the early stages but individual-specific taxa become more important in later stages of lactation.

*Pseudomonas* has been reported as a dominant member of the human milk microbiota in several studies including Ward *et al*., where it accounted for 61% of the relative abundance from milk samples taken between 9 and 30 days postpartum^14^,^21^,^22^. Similarly *Staphylococcus* has been found to be a common constituent of the milk microbiota by both culture independent and dependent investigations^11^,^14^,^15^,^21^,^22^,^23^. In a study of breast-fed Swedish infants, both vaginally and caesarean section delivered, 100% of infant feces were colonised by *Staphylococcus* from day 3 of life. *Staphylococcus epidermis* in particular appears to have a biological relevance as it has been shown to be the predominant species in human milk and in the faeces of breast-fed infants and is less common in stool of formula-fed infants^11^,^24^,^25^. Genera associated with the oral cavity such as *Streptococcus* and *Veillonella*’ reported by Hunt *et al*. and Cabrera-Rubio *et al*. were also prevalent in this study^14^,^15^. Although not previously reported in the human milk microbiome, *Variovorax,* strains of which have been isolated from the human oral cavity, were consistently detected in all samples^26^.

The detection of large proportions of typical inhabitants of the skin and oral microbiota may imply that the origin in this case is secondary contamination. However, anerobic gut-associated populations such as *Bacteroides, Blautia, Faecalibacterium, Ruminococcus, Roseburia, Subdoligranulum, Enterococcus* and *Escherichia-Shigella* were also detected here and in other studies^15^,^21^. *Bifidobacterium* was also consistently detected, supporting the findings of Jost *et al*., and Hunt *et al*., however Cabrera-Rubio *et al*. and Ward *et al*. did not report the presence of *Bifidobacterium.* These differences are likely due to the method of DNA extraction used, as a bead-beating step was not incorporated in the latter studies. When making comparisons across studies, it is important to note that differences in microbial community composition may also have been affected by factors such as the hypervariable region of the 16S gene examined (we targeted the V3–V4 hypervariable regions of 16S rDNA), geographical differences and the greater sequencing depth achieved using Illumina MiSeq sequencing here compared with Roche 454 pyrosequencing used in other studies. These factors are known to influence diversity and richness estimates and can greatly impact the microbiome of individuals.

When considering the infant fecal microbiota, it was found to be less diverse than that of human milk. The communities were most similar with respect to *Staphylococcus* which accounted for a mean relative abundance of 15% and 19% at week 1 in human milk and infant faeces, respectively. A number of typically gut-associated genera were common to both human milk and infant faeces including *Bifidobacterium, Bacteroides, Enterococcus, Lactobacillus, Clostridium, Coprococcus, Escherichia-Shigella* and members of the Lachnospiraceae family. Interestingly, these shared genera accounted for 70–88% of the total reads in infant faecal samples throughout the sampling period. This is in agreement with other studies in mother-infant pairs which have shown that the bacterial composition of the faecal microbiota of the breast-fed infants reflects that found in the breast milk^12^,^24^,^27^,^28^,^29^,^30^. In these studies, the genera *Lactobacillus, Staphylococcus, Enterococcus* and *Bifidobacterium* were frequently shared between breast milk and infant faeces. Jimenez *et al*., in a study with 23 mother-infant pairs identified *Staphylococcus* as the predominant species in milk and breast-fed infants faeces^25^. Gronlund *et al*., reported that maternal breast-milk bifidobacterial counts impacted on the infants’ faecal *Bifidobacterium* levels^27^. While *Jost et al*., identified a number of gut-associated anaerobic genera like *Bifidobacterium, Bacteroides* and members of the class Clostridia shared between milk and infant faeces^30^.

Stronger evidence of vertical transfer from mother to infant involves identification of identical strains. *Lactobacillus* and *Bifidobacterium* are often considered as members of a healthy microbiota with the predominance of *Bifidobacterium* in particular appearing to be characteristic of the healthy breast-fed infant and were therefore targeted for strain identification. Despite being detected by MiSeq sequencing, culturable *Lactobacillus* and *Bifidobacterium* were found in only one milk sample with a low bacterial count of 3 × 10^3^ CFU/ml and 1 × 10^2^ CFU/ml, respectively. Similarly, Albesharat *et al*., were unable to isolate any bifidobacteria from human milk samples^29^. It is unclear whether this was due to their low abundance in human milk, the presence of antimicrobial compounds, the length of storage time of samples prior to culturing and in the case of bifidobacteria, their anaerobicity i.e. viable but not culturable. As MiSeq sequencing does not distinguish between live and dead cells, it is also possible that dead cells are being transferred during feeding which would nonetheless elicit an immune response. Of interest in this case was that these culturable isolates belonged to the same species as those isolated from the corresponding infant fecal samples, namely *L. plantarum* and *B. breve*. PFGE analysis confirmed that the same bacterial strain was shared in this mother-infant pair supporting the notion of vertical transfer via human milk. Other studies have also isolated the same *Bifidobacterium* and *Lactobacillus* strains from human milk and infant faeces including strains of *B. breve* and *L. plantarum*^13^,^28^.

A limitation of this study is the relatively small sample size and further investigations in larger populations, including maternal faecal samples are planned to confirm these results and extend the knowledge about the milk microbiome. In summary, our data demonstrate the large diversity of the human milk microbiota with over 207 bacterial genera identified. The relative abundances of these were unique to each mother and subject to variation over time. Coupled with our finding that the same strains of *Bifidobacterium* and *Lactobacillus* were found in maternal human milk and corresponding infant fecal samples, these results suggest that there is a microbiota specific for each mother-infant pair that could confer benefits specific to that infant. This is particularly pertinent as commercially available infant formulas and donor milk are sterilized/pasteurised and as such contain little to no microbes. The results of this work also confirmed the presence of microbes typically associated with the gut microbiota in milk samples suggesting strategies to manipulate the maternal gut with bacteria which could confer benefits to the infant may be beneficial. These data emphasise that human milk constitutes a relevant source of a wide range of bacteria for the infant gut and can contribute to infant gut colonisation and therefore to infant health.

## Methods

### Subjects, Study Design and Sample Collection

This study was approved by the Clinical Research Ethics Committee of the Cork Teaching Hospitals. Parents of infant participants provided written informed consent and all relevant guidelines and regulations were followed. Ten mother-infant pairs were recruited at Cork University Maternity Hospital. Recruits were healthy lactating women and their full-term, healthy breast-fed infants (breast-fed for a minimum of 4 weeks after birth). The clinical characteristics of mother-infant pairs are shown in Table 2. Milk and faecal samples were collected from each mother-infant pair at 1, 3, 6 and 12 weeks. For milk sampling, sterile gloves were worn and the nipple and areola of the human were cleaned with chlorhexidane wipes (Clinell, United Kingdom) prior to manual expression of human milk into a sterile tube. The first few drops (approximately 1 ml) were discarded to prevent chlorhexidine contamination. Faecal samples were also collected into sterile containers and stored at 4 °C until delivery to the laboratory. Aliquots of 1 ml of fresh milk and 1 g faecal sample were subject to culture within 24 hours of donation. Remaining aliquots were immediately frozen at −20 °C for subsequent DNA extraction.

### DNA Extraction and MiSeq Sequencing

DNA was purified from stool samples using the QIAmp DNA Stool Mini Kit (Qiagen, UK) according to manufacturer’s instructions with the addition of a bead-beating step (30 s × 3). For milk samples, 1 ml was centrifuged at 5,000 g for 30 minutes. The supernatant was removed and any residue removed with a sterile cotton swab. The pellet was washed twice with sterile PBS and incubated at 55 °C for 15 minutes with 50 mg/ml lysozyme and 5 KU/ml mutanolysin. The remaining steps were performed using the QIAmp DNA Stool Mini Kit according to manufacturer’s instructions with addition of a bead-beating step (30 s × 3). The microbiota composition of the samples was established by amplicon sequencing of a ~380 bp fragment of the V3-V4 hypervariable region of the bacterial 16S rRNA gene following the Illumina 16S Metagenomic Sequencing Library Preperation guide. PCR amplification of V3–V4 region was performed using the forward primer 5′TCGTCGGCAGCGTCAGATGTGTATAAGAGACAGCCTACGGGNGGCWGCAG and reverse primer 5′GTCTCGTGGGCTCGGAGATGTGTATAAGAGACAGGACTACHVGGGTATCTAATCC. Each 25 μl PCR reaction contained 5 ng/μl microbial genomic DNA, 1 μM of each primer and 12.5 μl 2× KAPA HiFi HotStart ReadyMix. The PCR conditions for faecal DNA were as follows: initial denaturation for 5 min at 95 °C; 30 cycles of 15 s at 95 °C, 15 s at 42 °C and 30 s at 72 °C; and 72 °C for 5 min for final extension. The PCR conditions for milk DNA were: initial denaturation for 5 min at 95 °C; 30 cycles of 15 s at 95 °C, 15 s at 57 °C and 30 s at 72 °C; and 72 °C for 5 min for final extension. The Agencourt AMPure XP system (Beckman Coulter, UK) was used to purify the amplicons. A subsequent limited‐cycle amplification step was performed to add multiplexing indices and Illumina sequencing adapters. Amplicons were quantified, normalised and pooled using the Qubit^®^ dsDNA HS Assay Kit (Life Technologies). Library preparation was carried out by GATC Biotech prior to 2 × 300 base-pair (bp) sequencing on the Illumina MiSeq platform.

### Sequence and Statistical Analysis

300 bp paired-end reads were assembled using FLASH with parameters of a minimum overlap of 20 bp and a maximum overlap of 120 bp^31^. The QIIME suite of tools, v1.8.0, was used for further processing of paired-end reads, including quality filtering based on a quality score of >25 and removal of mismatched barcodes and sequences below length thresholds^32^. Denoising, chimera detection and operational taxonomic unit (OTU) grouping were performed in QIIME using USEARCH v7^33^. Taxonomic ranks were assigned by alignment of OTUs using PyNAST to the SILVA SSURef database release 111^34^,^35^. Alpha and beta diversities were generated in QIIME and calculated based on weighted and unweighted Unifrac distance matrices^36^. Principal coordinate analysis (PCoA) plots were visualised using EMPeror v0.9.3-dev^37^. To determine any statistically significant differences in microbial diversity between milk and faecal samples, non-parametric Mann-Whitney analysis was completed using Minitab 15 statistical software package. Statistical significance was accepted as p < 0.05, adjusted for ties.

### Isolation and Enumeration of *Bifidobacterium* and *Lactobacillus* spp

Aliquots of faecal sample, 1 gram, were mixed with 9 ml maximum recovery diluent (Oxoid) to make an initial 10^−1^ dilution. Serial dilutions were spread-plated onto de Man, Rogosa, Sharpe (MRS) (Difco) agar supplemented with 0.05% L-cysteine hydrochloride (Sigma), 100 μg ml^−1^ mupirocin (Oxoid) and 50 Units nystatin (Sigma Aldrich) for *Bifidobacterium* spp. and MRS agar (Difco) supplemented with 50 U ml^−1^ nystatin for *Lactobacillus* spp. 1 ml of milk was serially diluted and spread-plated as described above. Agar plates were incubated anaerobically at 37 °C for 72 hours for *Bifidobacterium* spp. and 5 days for *Lactobacillus* spp. Bacterial counts were recorded as colony forming units per gram of faeces or per ml of human-milk.

### Species Determination by Sanger Sequencing

Bacterial isolates from the *Bifidobacterium*-selective and *Lactobacillus*-selective media were grown overnight in MRS broth and genomic DNA extracted using the GenElute™ Bacterial Genomic DNA kit (Sigma) following manufacturer’s instructions. The identity of each putative *Bifidobacterium* isolate was confirmed by generating a 1.5 kb 16s rRNA gene-internally transcribed spacer (ITS) fragment using the oligonucleotides BIF-specific (5′-GGTGTGAAAGTCCATCGCT-3′) and 23S_bif (5′-GTCTGCCAAGGCATCCACCA-3′)^38^. Each 50 μl PCR reaction contained 45 μl of Platinum PCR SuperMix High Fidelity (Invitrogen), 1 μl BIF-specific primer (0.15 μM), 1 μl 23S_bif primer (0.15 μM), template DNA and sterile PCR grade water. PCRs were completed under the following conditions: initial denaturation step of 3 min at 94 °C, followed by 35 cycles of 30 s at 94 °C, 30 s at 56.5 °C and 1 min at 72 °C, followed by a single elongation step of 10 min at 72 °C. The resulting amplicons were separated on 1% (w/v) agarose gels and purified using the QIAquick PCR purification kit (Qiagen, United Kingdom). DNA sequencing of both strands was performed by Beckman Coulter (Essex, UK) using the oligonucleotides bif-sec (5′-CATGCCCCTACGTCCAG-3′) and 23S-bif (5′-CAAGGCATCCACCATACGC-3′). Strains were assigned to a particular species following comparison of the 16s rRNA-ITS sequences using NCBI BLAST database (http://www.ncbi.nlm.nih.gov/BLAST/).

### Genetic Typing by Pulsed-Field Gel Electrophoresis (PFGE)

High-molecular-weight DNA fragments were isolated from stationary phase cultures by a previously described method^39^. The restriction enzyme XbaI was used to cleave bifidobacterial chromosomal DNA and ApaI (New England Biolabs, MA, United States) was used for *Lactobacillus*. The fragments were separated using a contour-clamped homogeneous electric field CHEF-DR III pulsed field system (Bio-Rad Laboratories) with a linear ramp pulse time of 1 to 15 s and 2 to 20 s for bifidobacteria and lactobacilli, respectively. The run time was 18 h at 6 V/cm in a running buffer of 0.5X Tris base-borate-EDTA maintained at a temperature of 12 °C.

## Additional Information

**How to cite this article:** Murphy, K. *et al*. The Composition of Human Milk and Infant Faecal Microbiota Over the First Three Months of Life: A Pilot Study. *Sci. Rep.* **7**, 40597; doi: 10.1038/srep40597 (2017).

**Publisher's note:** Springer Nature remains neutral with regard to jurisdictional claims in published maps and institutional affiliations.

## Supplementary Material

Supplementary Information

## Figures and Tables

**Table 1:** Shared genera between human milk and infant faeces at each week as average percentage relative abundances and p-values.

	Milk Week 1	Faeces Week 1	p-value	Milk Week 3	Faeces Week 3	p-value	Milk Week 6	Faeces Week 6	p-value	Milk Week 12	Faeces Week 12	p-value
Actinobacteria												
*Actinomyces*				0.01	0.07	ns	0.01	0.26	ns	0.05	0.60	0.0384
*Bifidobacterium*	2.67	9.68	ns	1.86	18.20	0.0433	1.07	12.67	0.0206	1.54	13.82	0.0370
*Atopobium*							0.02	0.03	ns	0.02	0.01	ns
*Corynebacterium*				1.18	0.02	ns	0.18	0.02	ns	0.45	0.01	ns
*Rhodococcus*							0.05	0.02	ns	0.14	0.11	ns
*Rothia*				0.01	0.17	0.0216				0.18	0.02	ns
*Propionibacterium*							0.11	0.06	ns	0.31	0.02	ns
Bacteroidetes												
*Bacteroides*	1.06	1.37	ns				0.13	6.77	0.0271			
*Prevotella*										0.06	0.01	ns
Firmicutes												
*Gemella*	1.18	0.07	ns	2.20	0.05	ns	0.32	0.04	ns			
*Staphylococcus*	12.44	18.91	ns	5.57	7.27	ns	6.90	2.62	ns	1.33	0.15	Ns
*Granulicatella*	0.04	0.01	ns				0.15	0.01	ns			
*Enterococcus*	0.59	0.71	ns	1.52	0.15	0.0293	0.13	1.57	ns	0.16	4.47	0.0005
*Lactobacillus*	2.56	6.15	0.0038	2.49	7.20	ns	2.99	6.43	ns	2.02	2.20	ns
*Streptococcus*	10.17	11	ns	31.58	13.85	ns	5.67	14.80	ns	6.46	5.98	ns
*Anaerococcus*										0.05	0.08	ns
*Finegoldia*										0.21	0.08	ns
*Peptoniphilus*										0.06	0.03	ns
*Coprococcus*										0.19	0.07	ns
Lachnospiraceae incertae sedis	0.46	1.25	ns	0.15	0.71	0.0398	0.20	2.49	ns	1.34	6.92	ns
Peptostreptococcaceae incertae sedis							0.03	0.29	ns			
*Veillonella*	0.98	13.66	0.0369	1.94	14.69	ns	0.24	22.73	0.0011	1.32	10.47	0.0109
Erysipelotrichaceae incertae sedis	0.23	2.54	ns	0.13	1.32	ns	0.13	0.34	ns	0.46	0.67	ns
Proteobacteria												
*Citrobacter*	0.21	4.35	0.0065	0.38	1.08	0.0019	0.12	3.71	ns	0.18	0.79	ns
*Enterobacter*	1.87	1.24	0.0010	1.74	1.36	0.002	1.95	3.85	0.0290	2.93	7.14	ns
*Erwina*							0.09	0.03	ns			
*Escherichia-Shigella*	0.03	11.81	0.0171	0.48	17.28	ns	0.38	7.26	0.0182	0.17	20.65	0.0077
*Hemophilus*	2.77	3.82	ns	0.16	1.12	ns	0.02	0.87	ns	0.03	0.02	ns

**Table 2:** Clinical characteristics of the mothers and infants enrolled in this study.

Maternal pre-pregnancy BMI (kg)	24.7 [4.4]
Maternal height (cm)	164.5 [9.8]
Maternal weight (kg)	64.6 [5.9]
Birth weight (g)	3525 [510]
Gestational age (weeks)	39.1 [0.9]
Infant Gender	
*Female*	60%
*Male*	40%
Mode of delivery	
*SVD*	60%
*CS*	40%

The data for mothers and infants are shown as median values and interquartile ranges or as a percentage. SVD = Spontaneous vaginal delivery; CS = Caesarean section.
