# Supplementary material for: The Composition of Human Milk and Infant Faecal Microbiota Over the First Three Months of Life: A Pilot Study
Source: Sci Rep. 2017 Jan 17;7:40597. doi: 10.1038/srep40597 (PMC5240090; doi:10.1038/srep40597)
Supplement: Supplementary Information [file srep40597-s1.doc]

# SUPPLEMENTARY INFORMATION

**The Composition of Human Milk and Infant Faecal Microbiota Over the First Three Months of Life: A Pilot Study**

Kiera Murphy^1,2,3^, David Curley^4^, Tom F O’ Callaghan^1,2^ , Carol-Anne O’Shea^3,4^, Eugene M Dempsey^3,4^, Paul W O’ Toole^2,3^, R Paul Ross^3,5^, C Anthony Ryan^3,4^ and Catherine Stanton^1,3*^.

1. Food Biosciences, Teagasc Food Research Centre, Fermoy, Co Cork, Ireland;

2. School of Microbiology, University College Cork, Cork, Ireland;

3. APC Microbiome Institute, University College Cork, Cork, Ireland;

4. Department of Neonatology, Cork University Maternity Hospital, Cork, Ireland;

5. College of Science, Engineering and Food Science, University College Cork, Cork, Ireland.

* Corresponding Author: [catherine.stanton@teagasc.ie](mailto:catherine.stanton@teagasc.ie)

**Supplementary Table S1: Relative abundance (%) of the core microbiota at genus level and remaining taxa for each mother, M1 – M10, at week 1, 3, 6 and 12.**

* = Sample not collected.

|  | **M1-W1** | **M1-W3*** | **M1-W6** | **M1-W12** |
| --- | --- | --- | --- | --- |
| **Core** | **68.52** |  | **78.41** | **67.25** |
| *Telmatobacter* |  |  |  | 0.37 |
| *Iamia* |  |  |  | 0.26 |
| *Actinomyces* |  |  |  | 0.08 |
| *Atopobium* |  |  |  | 0.14 |
| *Mycobacterium* |  |  |  | 0.33 |
| *Humicoccus* |  |  |  | 0.14 |
| *Brevibacterium* |  |  |  | 0.02 |
| *Dermacoccus* |  |  |  | 0.13 |
| *Rhodoluna* | 0.20 |  |  |  |
| *Microbacterium* | 0.58 |  |  | 0.17 |
| *Kocuria* | 0.52 |  |  | 0.01 |
| *Nesterenkonia* | 1.85 |  |  |  |
| *Bacteroides* | 0.55 |  |  |  |
| *Cloacibacterium* | 0.07 |  | 0.11 |  |
| *Corynebacterium* |  |  | 0.18 |  |
| *Rhodococcus* |  |  | 0.06 |  |
| *Micrococcus* |  |  |  | 0.31 |
| *Nocardioides* |  |  |  | 0.16 |
| *Propionibacterium* |  |  | 0.03 | 0.66 |
| Chitinophagaceae uncultured |  |  |  | 0.29 |
| *Pseudonocardia* |  |  |  | 0.13 |
| *Patulibacter* |  |  |  | 0.53 |
| *Prevotella* |  |  |  | 0.19 |
| *Alistipes* |  |  |  | 0.07 |
| *Chryseobacterium* |  |  |  | 3.58 |
| *Pedobacter* | 0.13 |  | 0.50 | 2.15 |
| *Sphingobacterium* | 0.07 |  |  |  |
| Candidate division OD1 uncultured | 2.12 |  | 1.53 | 1.35 |
| Candidate division OP11 uncultured |  |  | 0.33 | 0.19 |
| Candidate division TM7 uncultured | 0.19 |  | 0.35 | 0.15 |
| *Bacillus* | 3.43 |  |  |  |
| Anaerolineaceae uncultured |  |  | 0.10 |  |
| Chloroflexi uncultured |  |  | 0.77 | 0.05 |
| Cyanobacteria uncultured |  |  | 1.22 | 0.58 |
| Fibrobacteraceae uncultured |  |  |  | 0.23 |
| *Anoxybacillus* |  |  | 0.57 | 0.39 |
| *Gemella* | 0.05 |  |  |  |
| *Aerococcus* | 1.81 |  |  |  |
| *Exiguobacterium* |  |  |  | 0.37 |
| *Planomicrobium* |  |  |  | 0.02 |
| *Leuconostoc* |  |  |  | 0.26 |
| *Granulicatella* | 0.09 |  |  |  |
| *Lactococcus* | 0.40 |  | 0.11 | 0.08 |
| *Anaerococcus* |  |  |  | 0.37 |
| *Finegoldia* |  |  |  | 1.65 |
| *Helcococcus* |  |  |  | 0.02 |
| *Peptoniphilus* |  |  |  | 0.52 |
| *Anaerostipes* |  |  |  | 0.65 |
| *Coprococcus* |  |  |  | 0.66 |
| Lachnospiraceae Incertae Sedis |  |  |  | 1.00 |
| Lachnospiraceae uncultured | 0.24 |  |  | 1.08 |
| *Roseburia* |  |  | 0.66 |  |
| *Fastidiosipila* |  |  |  | 0.14 |
| *Dialister* |  |  |  | 0.37 |
| *Veillonella* | 3.72 |  | 0.01 | 0.01 |
| *Fusobacterium* | 1.01 |  |  |  |
| Erysipelotrichaceae Incertae Sedis |  |  | 0.48 | 2.16 |
| *Devosia* |  |  | 1.20 |  |
| *Aminobacter* |  |  | 0.01 |  |
| *Ochrobactrum* | 0.82 |  | 0.01 |  |
| *Phyllobacterium* |  |  | 0.53 |  |
| Phyllobacteriaceae uncultured |  |  | 0.29 |  |
| *Leptotrichia/Sneathia* |  |  |  | 0.30 |
| *Rhizobium* |  |  | 2.49 | 2.79 |
| *Paracoccus* |  |  | 0.60 |  |
| *Methylobacterium* | 0.17 |  |  |  |
| Rhodospirillaceae uncultured |  |  |  | 0.57 |
| *Sphingomonas* | 0.33 |  |  | 1.16 |
| *Achromobacter* | 1.84 |  | 3.01 | 0.41 |
| *Ralstonia* | 0.01 |  | 3.22 | 0.88 |
| *Acidovorax* | 4.13 |  | 0.32 | 0.52 |
| *Aquabacterium* | 0.09 |  |  |  |
| *Curvibacter* |  |  | 0.74 | 0.22 |
| *Limnohabitans* | 0.01 |  | 0.01 | 0.14 |
| *Massilia* | 2.28 |  |  |  |
| *Pseudorhodoferax* |  |  |  | 0.18 |
| Comamonadaceae uncultured |  |  | 0.19 | 0.07 |
| *Undibacterium* | 0.39 |  | 0.07 |  |
| Methylophilaceae uncultured | 0.02 |  |  |  |
| *Nitrosococcus* |  |  | 0.83 |  |
| *Bilophila* |  |  |  | 0.28 |
| *0319-6G20* uncultured |  |  |  | 0.17 |
| *Shewanella* |  |  |  | 0.16 |
| *Citrobacter* | 0.21 |  | 0.13 | 0.17 |
| *Erwinia* |  |  | 0.01 |  |
| *Escherichia-Shigella* |  |  |  | 0.16 |
| *Haemophilus* |  |  | 0.17 | 0.06 |
| *Acinetobacter* | 0.63 |  | 0.41 | 0.31 |
| *Pseudoxanthomonas* | 1.87 |  |  |  |
| *Chthoniobacter* | 0.12 |  |  |  |
| *Enhydrobacter* |  |  |  | 0.24 |
| *Akkermansia* |  |  |  | 0.26 |

|  | **M2-W1** | **M2-W3** | **M2-W6** | **M2-W12** |
| --- | --- | --- | --- | --- |
| **Core** | **85.06** | **81.10** | **83.93** | **50.02** |
| *Corynebacterium* | 0.09 |  | 0.12 |  |
| *Leifsonia* | 0.15 |  |  |  |
| *Bryobacter* |  |  |  | 0.10 |
| *Iamia* |  |  |  | 0.25 |
| *Actinomyces* |  |  |  | 0.16 |
| *Microbacterium* | 0.51 |  |  | 0.04 |
| *Micrococcus* |  |  |  | 0.30 |
| *Sphingobacterium* | 0.01 |  |  |  |
| *Rothia* |  |  |  | 0.32 |
| *Propionibacterium* |  | 0.31 |  | 0.23 |
| *Yaniella* |  |  | 0.03 |  |
| *Prevotella* |  |  |  | 0.13 |
| *Chryseobacterium* |  |  |  | 1.13 |
| *Pedobacter* |  |  | 2.65 | 0.56 |
| Candidate division OD1 uncultured | 0.98 | 1.44 | 2.49 | 1.34 |
| *Candidate division OP11* uncultured |  |  |  | 0.31 |
| Cyanobacteria uncultured | 0.04 |  |  |  |
| *Gemella* |  |  |  |  |
| *Cyanobacteria* uncultured |  |  |  | 0.49 |
| *Anoxybacillus* |  | 0.10 |  | 0.14 |
| *Enterococcus* | 1.96 | 3.28 |  |  |
| *Blautia* |  | 0.10 |  |  |
| *Coprococcus* |  |  | 0.09 |  |
| *Abiotrophia* |  |  |  | 0.05 |
| *Facklamia* |  |  |  | 0.55 |
| *Granulicatella* |  |  |  | 0.30 |
| Lachnospiraceae Incertae Sedis | 1.41 | 0.95 | 0.08 | 0.01 |
| Lachnospiraceae uncultured |  | 0.21 |  | 0.47 |
| Peptostreptococcaceae Incertae Sedis | 0.56 |  |  |  |
| *Ruminococcus* |  | 1.39 |  |  |
| Erysipelotrichaceae Incertae Sedis | 0.13 | 0.40 |  |  |
| *Selenomonas* |  |  |  | 0.10 |
| *Veillonella* |  |  |  | 1.46 |
| *Fusobacterium* |  |  |  | 0.01 |
| *Leptotrichia/Sneathia* |  |  |  | 0.09 |
| *Ochrobactrum* | 0.01 | 0.01 |  | 0.27 |
| *Bosea* |  |  | 0.05 |  |
| *Devosia* | 0.02 |  | 0.01 | 0.47 |
| *Methylobacterium* |  |  |  |  |
| *Phyllobacterium* | 1.76 | 1.99 |  | 0.90 |
| Phyllobacteriaceae uncultured |  | 0.10 |  |  |
| *Rhizobium* | 0.65 | 2.24 | 3.19 | 2.38 |
| *Paracoccus* |  |  |  | 0.02 |
| *Sphingobium* |  |  |  | 1.27 |
| *Sphingomonas* | 0.79 |  | 0.08 | 0.34 |
| *Ralstonia* |  | 0.43 | 1.59 | 1.40 |
| *Achromobacter* | 0.17 | 0.74 |  | 1.23 |
| *Burkholderia* | 0.01 |  |  | 0.13 |
| *Acidovorax* | 0.14 | 0.47 | 0.34 |  |
| *Curvibacter* |  |  |  | 0.04 |
| *Limnohabitans* | 0.02 | 0.82 | 1.27 | 0.60 |
| *Pelomonas* |  | 0.09 |  |  |
| *Polaromonas* |  |  |  | 0.10 |
| *Pseudorhodoferax* |  | 0.02 | 0.04 | 0.02 |
| Comamonadaceae uncultured |  | 0.01 | 0.01 | 0.09 |
| *Massilia* |  | 1.67 |  |  |
| *Undibacterium* | 0.17 | 0.10 | 0.74 |  |
| *Neisseria* |  |  |  | 0.72 |
| *Citrobacter* | 0.04 | 0.30 | 0.20 | 0.08 |
| *Escherichia-Shigella* | 0.01 | 0.06 | 2.58 |  |
| *Actinobacillus* |  |  |  | 0.11 |
| *Haemophilus* |  |  |  | 0.10 |
| *Acinetobacter* |  | 0.03 |  | 31.70 |
| *Akkermansia* |  |  | 0.38 |  |

|  | **M3-W1** | **M3-W3** | **M3-W6** | **M3-W12** |
| --- | --- | --- | --- | --- |
| **Core** | **64.54** | **90.17** | **60.97** | **80.62** |
| *Gardnerella* | 0.01 | 0.03 |  |  |
| *Corynebacterium* |  | 0.06 |  |  |
| *Yaniella* |  | 0.10 |  |  |
| *Marmoricola* |  | 0.01 |  |  |
| *Blastococcus* |  |  | 0.58 |  |
| *Agrococcus* |  |  | 0.09 |  |
| *Leifsonia* |  |  | 0.16 |  |
| *Rhodococcus* |  |  |  | 1.12 |
| *Microbacterium* |  |  | 0.20 | 0.08 |
| *Propionibacterium* | 0.02 | 0.06 | 0.63 |  |
| *Bacteroides* | 4.50 |  | 1.00 |  |
| *Mucilaginibacter* | 2.56 |  |  |  |
| *Chryseobacterium* |  |  |  | 3.36 |
| *Cloacibacterium* |  | 0.23 |  |  |
| *Prevotella* |  |  | 1.30 |  |
| env.OPS 17 uncultured |  |  | 1.07 |  |
| *Pedobacter* | 3.95 | 0.48 | 3.12 | 1.36 |
| Candidate division OD1 uncultured | 1.89 | 0.04 | 1.50 | 1.66 |
| *Candidate division OP11* uncultured |  |  | 0.84 |  |
| Candidate division TM7 uncultured | 0.26 |  | 0.65 |  |
| Chloroflexi uncultured | 0.31 | 0.01 | 0.78 |  |
| Cyanobacteria uncultured | 1.50 |  | 0.64 | 0.16 |
| *Bacillus* | 1.27 |  | 1.19 |  |
| *Anoxybacillus* |  | 0.35 |  |  |
| *Gemella* |  | 2.16 |  | 0.53 |
| *Alkalibacterium* |  | 0.12 |  |  |
| *Granulicatella* |  | 0.03 |  |  |
| *Planomicrobium* |  |  | 2.00 | 0.01 |
| *Carnobacterium* |  |  | 0.28 |  |
| *Enterococcus* |  | 0.12 | 0.71 |  |
| *Blautia* |  | 0.01 | 0.52 |  |
| Lachnospiraceae Incertae Sedis | 0.01 | 0.03 | 1.25 | 2.25 |
| Lachnospiraceae uncultured |  |  | 0.01 | 0.03 |
| Peptostreptococcaceae Incertae Sedis |  |  | 0.25 |  |
| Peptostreptococcaceae uncultured |  |  | 0.05 |  |
| *Faecalibacterium* | 2.61 |  | 1.39 |  |
| *Subdoligranulum* | 4.12 |  | 0.03 |  |
| Ruminococcaceae uncultured | 3.93 |  | 0.02 |  |
| *Phyllobacterium* | 0.10 | 0.15 |  |  |
| *Veillonella* |  | 1.48 |  |  |
| Erysipelotrichaceae *Incertae Sedis* |  | 0.07 | 0.53 |  |
| *Gemmatimonas* |  | 0.01 | 0.28 | 0.26 |
| *Rhizobium* | 1.55 | 0.23 |  |  |
| Rhodospirillaceae uncultured | 1.19 |  |  |  |
| Planctomycetaceae uncultured |  |  | 0.88 |  |
| *Devosia* |  |  | 0.26 | 0.30 |
| *Methylobacterium* |  |  | 0.67 | 0.65 |
| *Geminicoccus* |  |  | 0.48 |  |
| Erythrobacteraceae uncultured |  |  | 0.75 |  |
| *Rhizobium* |  |  | 3.13 | 2.18 |
| *Sphingobium* |  |  | 0.30 | 0.01 |
| *Sphingomonas* |  |  | 0.75 |  |
| *Sphingopyxis* |  |  | 0.46 |  |
| *Paracoccus* |  | 0.06 | 2.46 |  |
| *Achromobacter* |  | 0.03 | 1.41 | 2.57 |
| *Ralstonia* | 1.15 | 0.31 | 1.84 | 3.10 |
| *Acidovorax* |  | 0.01 |  |  |
| *Limnohabitans* | 0.02 |  | 0.19 | 0.83 |
| *Pseudorhodoferax* |  |  |  | 0.06 |
| Comamonadaceae uncultured | 0.04 |  |  | 0.36 |
| *Collimonas* |  |  |  | 0.79 |
| *Massilia* |  |  |  |  |
| *Undibacterium* | 2.55 |  |  | 0.18 |
| *Polaromonas* |  | 0.01 | 0.37 |  |
| Comamonadaceae uncultured |  |  | 0.04 |  |
| *Aquaspirillum* |  | 0.01 |  |  |
| *Neisseria* |  | 0.13 |  |  |
| *Bdellovibrio* | 0.05 | 0.01 | 0.02 |  |
| *Bilophila* |  |  | 1.42 |  |
| *Azovibrio* |  |  |  | 0.33 |
| *Syntrophorhabdus* |  |  |  | 0.21 |
| *Citrobacter* | 0.07 | 1.55 | 0.16 | 0.10 |
| *Serratia* |  |  | 0.01 |  |
| *Aquicella* |  |  | 0.35 |  |
| *Escherichia-Shigella* | 0.01 | 0.06 |  |  |
| *Piscirickettsia* | 0.36 |  |  |  |
| *Pseudofulvimonas* | 0.06 |  |  |  |
| *Acinetobacter* |  | 0.67 | 0.37 |  |
| *Lysobacter* |  |  | 0.44 |  |

|  | **M4-W1** | **M4-W3** | **M4-W6** | **M4-W12** |
| --- | --- | --- | --- | --- |
| **Core** | **82.89** | **78.72** | **87.75** | **74.24** |
| *Corynebacterium* | 1.01 |  |  |  |
| *Mycobacterium* | 0.07 |  |  |  |
| *Actinomyces* |  |  | 0.01 | 0.12 |
| *Atopobium* |  |  | 0.20 |  |
| *Rhodococcus* |  |  | 0.24 |  |
| *Collinsella* |  |  |  | 0.04 |
| *Leucobacter* |  |  |  | 0.38 |
| *Microbacterium* | 0.01 | 0.01 | 0.45 | 0.10 |
| Chitinophagaceae uncultured | 0.99 |  |  |  |
| *Rothia* |  |  |  | 0.65 |
| *Yaniella* |  |  |  | 0.37 |
| *Propionibacterium* |  |  | 0.13 | 0.43 |
| *Solirubrobacter* |  |  |  | 0.10 |
| *Alistipes* |  |  |  | 0.09 |
| S24-7 uncultured |  |  |  | 0.49 |
| *Capnocytophaga* |  |  |  | 0.57 |
| *Chryseobacterium* |  |  |  | 1.84 |
| *Wautersiella* |  |  |  | 0.12 |
| *Pedobacter* | 0.98 | 0.08 | 0.03 | 1.16 |
| *Sphingobacterium* | 0.17 |  |  | 0.01 |
| Candidate division OD1 uncultured | 0.28 | 0.11 | 1.07 | 1.50 |
| Candidate division TM7 uncultured | 0.03 |  |  |  |
| Chloroflexi uncultured |  |  |  | 0.17 |
| Cyanobacteria uncultured | 0.36 |  | 0.16 |  |
| *Anoxybacillus* |  |  |  | 0.86 |
| *Gemella* | 3.50 | 0.48 | 1.15 | 0.06 |
| *Trichococcus* | 0.04 |  |  |  |
| *Enterococcus* | 0.58 |  | 0.12 | 0.12 |
| *Anaerostipes* |  |  |  | 0.15 |
| *Coprococcus* |  |  |  | 0.87 |
| Lachnospiraceae Incertae Sedis |  |  | 0.33 | 2.78 |
| Lachnospiraceae uncultured | 0.24 |  | 0.48 | 0.77 |
| Peptostreptococcaceae Incertae Sedis | 0.17 |  |  |  |
| *Selenomonas* |  |  |  | 0.59 |
| *Veillonella* | 0.13 | 0.52 | 1.84 | 4.03 |
| Erysipelotrichaceae Incertae Sedis | 0.13 |  |  | 0.14 |
| *Ochrobactrum* | 1.04 |  |  |  |
| *Devosia* | 0.01 |  |  |  |
| *Methylobacterium* | 0.01 |  |  |  |
| *Phyllobacterium* | 0.08 |  |  |  |
| Phyllobacteriaceae uncultured | 0.47 |  |  |  |
| Planctomycetaceae uncultured |  |  | 0.48 |  |
| *Gemmatimonas* |  |  |  | 0.09 |
| *Rhodopseudomonas* |  |  |  | 0.28 |
| *Rhizobium* | 0.69 | 0.04 | 0.55 | 0.77 |
| *Paracoccus* | 0.09 |  |  | 1.05 |
| *Sphingobium* |  | 0.11 |  |  |
| *Sphingomonas* |  |  |  | 0.22 |
| *Achromobacter* | 0.64 | 0.04 | 0.01 | 1.31 |
| *Ralstonia* | 1.32 | 0.11 | 0.90 | 0.97 |
| *Acidovorax* |  | 0.08 | 0.21 | 0.68 |
| *Comamonas* |  |  |  | 0.11 |
| *Limnohabitans* | 0.01 |  | 0.32 | 0.61 |
| *Pseudorhodoferax* |  |  |  | 0.03 |
| Comamonadaceae uncultured | 0.01 |  | 0.27 | 0.09 |
| *Undibacterium* | 0.01 |  | 0.52 | 0.06 |
| Methylophilaceae uncultured |  | 0.12 | 0.61 |  |
| *Azospira* |  |  | 0.65 |  |
| Sandaracinaceae uncultured |  |  |  | 0.21 |
| *Shewanella* |  |  |  | 0.32 |
| *Citrobacter* | 1.25 | 0.76 | 0.09 | 0.07 |
| *Erwinia* |  | 0.05 | 0.74 |  |
| *Escherichia-Shigella* | 0.16 |  | 0.01 | 0.40 |
| *Haemophilus* | 0.01 |  |  |  |
| *Acinetobacter* | 0.01 | 18.64 | 0.50 | 1.06 |
| *Enhydrobacter* |  |  | 0.02 |  |

|  | **M5-W1** | **M5-W3** | **M5-W6*** | **M5-W12** |
| --- | --- | --- | --- | --- |
| **Core** | **85.66** | **73.43** |  | **73.80** |
| *Corynebacterium* | 0.01 |  |  |  |
| *Rhodococcus* | 0.19 |  |  |  |
| *Leifsonia* | 0.03 |  |  |  |
| *Atopobium* |  | 0.11 |  |  |
| *Leucobacter* |  | 0.12 |  |  |
| *Microbacterium* |  | 0.04 |  | 0.11 |
| *Yaniella* |  | 0.27 |  |  |
| *Propionibacterium* | 0.03 | 0.07 |  |  |
| *Bergeyella* |  | 0.22 |  |  |
| *Kocuria* |  |  |  | 0.64 |
| *Chryseobacterium* |  |  |  | 3.36 |
| *Pedobacter* | 0.22 | 0.75 |  | 4.86 |
| Candidate division OD1 uncultured | 2.63 | 0.07 |  | 0.31 |
| *Chloroflexi* uncultured |  | 0.05 |  |  |
| Cyanobacteria uncultured | 0.46 |  |  | 0.11 |
| *Anoxybacillus* |  | 0.13 |  | 0.53 |
| *Gemella* | 1.75 | 3.32 |  |  |
| *Granulicatella* | 0.22 | 1.24 |  |  |
| *Enterococcus* | 0.03 | 0.20 |  |  |
| *Bacillus* |  |  |  | 2.57 |
| Lachnospiraceae Incertae Sedis | 0.44 | 0.12 |  | 1.00 |
| Lachnospiraceae uncultured | 0.24 | 0.04 |  |  |
| Veillonella | 0.72 | 13.54 |  | 0.01 |
| Erysipelotrichaceae Incertae Sedis | 0.01 | 0.06 |  |  |
| *Gemmatimonas* |  | 1.06 |  |  |
| *Ochrobactrum* | 0.18 | 0.12 |  | 0.05 |
| *Devosia* | 0.06 |  |  | 0.61 |
| *Phyllobacterium* |  |  |  | 1.65 |
| Phyllobacteriaceae uncultured | 0.01 |  |  |  |
| *Methylobacterium* |  | 0.33 |  |  |
| *Albibacter* |  | 0.06 |  |  |
| *Rhizobium* | 0.60 | 0.45 |  | 2.27 |
| Rhodospirillaceae uncultured | 0.57 |  |  | 0.36 |
| *Paracoccus* |  | 0.03 |  |  |
| *Novosphingobium* |  | 0.12 |  |  |
| *Achromobacter* | 0.01 | 0.19 |  | 0.26 |
| *Ralstonia* | 0.88 | 0.27 |  | 2.73 |
| *Acidovorax* | 2.01 | 0.01 |  | 1.88 |
| *Delftia* | 0.01 |  |  |  |
| *Curvibacter* |  | 0.01 |  |  |
| *Limnohabitans* | 0.02 | 0.03 |  | 0.01 |
| *Pelomonas* |  | 0.05 |  |  |
| Comamonadaceae uncultured | 0.04 | 0.04 |  | 0.19 |
| *Undibacterium* |  |  |  | 0.34 |
| *Neisseria* |  | 0.11 |  | 0.16 |
| *Bilophila* |  |  |  | 0.19 |
| *Citrobacter* | 0.13 | 0.03 |  | 0.41 |
| *Escherichia-Shigella* | 0.01 | 0.13 |  | 0.01 |
| *Haemophilus* | 0.18 | 0.79 |  | 0.01 |
| *Piscirickettsia* |  |  |  | 0.25 |
| *Acinetobacter* | 0.58 |  |  |  |

|  | **M6-W1** | **M6-W3** | **M6-W6** | **M6-W12** |
| --- | --- | --- | --- | --- |
| **Core** | **83.05** | **84.97** | **79.89** | **74.05** |
| *Chloracidobacterium* | 0.07 |  |  |  |
| *Actinomyces* | 0.01 |  |  |  |
| *Rhodococcus* | 0.02 |  |  |  |
| *Kocuria* | 0.31 |  |  |  |
| *Leifsonia* |  | 0.01 |  |  |
| *Microbacterium* |  | 0.09 |  |  |
| *Rothia* |  | 0.05 |  |  |
| *Microthrix* |  |  |  | 0.20 |
| *Yaniella* |  |  |  | 0.53 |
| *Propionibacterium* | 0.02 | 0.02 |  | 0.01 |
| *Bacteroides* | 0.02 |  |  |  |
| Rikenellaceae uncultured |  |  |  | 0.04 |
| *Chryseobacterium* |  |  |  | 2.95 |
| *Leifsonia* |  |  | 0.14 |  |
| *Pedobacter* | 0.60 | 0.46 | 1.77 | 2.01 |
| Candidate division OD1 uncultured | 0.08 | 0.31 | 3.40 | 3.35 |
| Candidate division TM7 uncultured |  |  | 0.02 | 1.67 |
| Cyanobacteria uncultured | 0.01 | 0.01 |  | 0.26 |
| *Anoxybacillus* | 0.87 |  | 0.39 | 2.39 |
| *Gemella* | 1.45 | 9.00 | 0.01 |  |
| *Granulicatella* | 0.01 |  |  |  |
| *Enterococcus* | 0.03 | 0.40 | 0.06 |  |
| Lachnospiraceae Incertae Sedis | 0.37 | 0.12 |  | 1.12 |
| Lachnospiraceae uncultured |  | 0.04 | 0.15 | 0.23 |
| *Roseburia* | 0.12 |  |  |  |
| *Veillonella* | 1.61 | 0.52 | 0.01 | 0.01 |
| *Gemmatimonas* | 0.01 |  |  |  |
| *Caulobacter* | 0.09 |  |  |  |
| *Devosia* | 0.01 | 0.26 | 1.23 |  |
| *Methylobacterium* | 0.01 | 0.21 |  |  |
| *Aminobacter* | 0.74 |  |  |  |
| Erysipelotrichaceae Incertae Sedis |  |  |  | 0.08 |
| *Ochrobactrum* |  |  |  | 0.53 |
| *Phyllobacterium* |  | 0.26 | 0.43 | 0.01 |
| Phyllobacteriaceae uncultured | 0.10 | 0.08 |  |  |
| *Rhizobium* | 0.14 | 0.39 | 1.68 | 3.80 |
| *Paracoccus* | 0.13 |  | 2.79 |  |
| Rhodospirillaceae uncultured | 0.17 |  |  |  |
| *Achromobacter* | 0.14 | 0.65 | 4.31 | 1.06 |
| *Ralstonia* | 0.90 | 0.12 | 2.38 | 0.50 |
| *Acidovorax* | 0.01 | 0.04 | 0.01 | 0.82 |
| *Curvibacter* | 0.34 |  |  | 0.14 |
| *Delftia* | 0.01 |  |  | 0.01 |
| *Limnohabitans* | 0.03 |  | 0.02 | 0.14 |
| *Schlegelella* |  |  | 0.37 |  |
| Comamonadaceae uncultured | 0.07 |  | 0.15 |  |
| *Massilia* | 0.01 |  |  |  |
| *Pseudorhodoferax* |  | 0.26 |  | 0.01 |
| *Bdellovibrio* |  |  | 0.59 |  |
| *Massilia* |  |  |  | 0.25 |
| *Citrobacter* | 0.04 | 0.01 | 0.12 | 0.17 |
| *Erwinia* |  |  |  | 0.01 |
| *Escherichia-Shigella* | 0.02 |  |  | 0.14 |
| *Haemophilus* | 0.48 | 0.62 |  |  |
| *Silanimonas* |  |  |  | 0.55 |
| *Lysobacter* |  | 0.15 |  |  |
| Sinobacteraceae uncultured | 0.29 |  |  |  |
| *Akkermansia* | 0.09 |  |  |  |

|  | **M7-W1** | **M7-W3** | **M7-W6** | **M7-W12** |
| --- | --- | --- | --- | --- |
| **Core** | **87.09** | **84.87** | **85.80** | **82.72** |
| *Bacteroides* | 0.02 |  |  |  |
| S24-7 uncultured | 0.28 |  |  |  |
| *Gardnerella* |  |  | 0.01 |  |
| *Corynebacterium* |  | 0.27 | 1.19 |  |
| *Agrococcus* |  |  |  | 0.01 |
| *Leifsonia* |  |  |  | 0.11 |
| *Microbacterium* |  |  |  | 0.48 |
| *Yaniella* |  | 0.08 |  | 0.47 |
| *Alpinimonas* |  |  | 0.96 |  |
| *Rhodoluna* |  |  | 0.43 |  |
| *Microbacterium* |  |  | 0.10 |  |
| *Propionibacterium* |  |  | 0.05 | 0.28 |
| *Bacteroides* |  |  |  | 0.01 |
| *Chryseobacterium* |  |  |  | 2.98 |
| *Pedobacter* | 2.67 | 0.35 | 0.04 | 2.19 |
| Candidate division OD1 uncultured | 0.48 | 1.01 | 3.01 | 0.40 |
| *Cyanobacteria* uncultured |  |  |  | 0.18 |
| *Gemella* |  |  | 0.01 |  |
| *Granulicatella* |  |  | 0.02 | 0.46 |
| *Enterococcus* | 1.49 | 2.51 | 0.13 |  |
| *Anaerostipes* | 0.11 |  |  |  |
| *Blautia* |  | 2.54 |  | 0.18 |
| Lachnospiraceae Incertae Sedis | 0.54 | 0.01 |  | 0.67 |
| Lachnospiraceae uncultured | 1.55 | 2.10 |  | 0.14 |
| Erysipelotrichaceae Incertae Sedis |  | 0.01 |  |  |
| *Caulobacter* |  | 0.01 |  |  |
| *Ochrobactrum* |  | 0.01 |  |  |
| *Devosia* | 0.59 | 0.12 |  |  |
| *Oscillibacter* |  |  |  | 0.28 |
| *Veillonella* |  |  | 0.03 | 3.08 |
| Erysipelotrichaceae Incertae Sedis |  |  |  | 0.26 |
| *Devosia* |  |  |  | 0.48 |
| *Phyllobacterium* | 1.93 | 0.72 | 0.07 | 0.10 |
| Phyllobacteriaceae uncultured |  |  | 0.15 |  |
| Rhizobium | 0.11 | 0.36 |  | 0.24 |
| Rhodospirillaceae uncultured |  | 0.06 | 1.27 |  |
| *Paracoccus* |  |  |  | 0.22 |
| *Achromobacter* | 0.26 | 1.01 | 1.39 | 1.17 |
| *Ralstonia* | 0.03 | 1.10 | 1.94 | 2.53 |
| *Acidovorax* | 0.22 | 0.57 | 0.86 | 0.61 |
| *Limnohabitans* | 1.20 |  | 0.04 | 0.62 |
| *Pseudorhodoferax* | 0.02 |  | 0.15 | 0.03 |
| *Delftia* |  | 0.01 |  |  |
| Comamonadaceae uncultured |  | 0.05 | 0.10 | 0.10 |
| *Undibacterium* | 0.01 | 0.26 | 0.55 |  |
| Methylophilaceae uncultured | 0.48 |  |  |  |
| *Citrobacter* |  | 0.32 | 0.14 | 0.33 |
| *Sinobacteraceae* uncultured |  | 0.13 |  |  |
| *Erwinia* |  |  |  | 0.01 |
| *Escherichia-Shigella* | 0.01 |  | 0.01 | 0.81 |
| *Akkermansia* |  |  | 0.01 |  |
| *Acinetobacter* |  |  |  | 0.37 |

|  | **M8-W1** | **M8-W3** | **M8-W6** | **M8-W12** |
| --- | --- | --- | --- | --- |
| **Core** | **81.07** | **81.71** | **75.92** | **80.93** |
| *Corynebacterium* |  |  | 0.01 |  |
| *Agrococcus* |  |  | 0.01 |  |
| *Microbacterium* | 0.31 |  | 0.61 |  |
| *Yaniella* | 0.30 |  |  |  |
| *Bacteroides* | 0.04 |  |  |  |
| *Parabacteroides* | 0.05 |  |  |  |
| *Lacibacter* | 0.01 |  |  |  |
| *Actinomyces* |  | 0.11 |  |  |
| *Propionibacterium* |  | 0.29 |  |  |
| *Chryseobacterium* |  |  |  | 5.47 |
| *Pedobacter* | 0.11 | 1.93 | 4.38 | 1.16 |
| Candidate division OD1 uncultured | 2.35 | 0.86 | 0.96 | 1.11 |
| Candidate division TM7 uncultured |  | 0.02 |  |  |
| Cyanobacteria uncultured |  |  | 2.52 |  |
| *Anoxybacillus* |  | 1.12 |  |  |
| *Bacillus* | 0.01 | 0.91 |  |  |
| *Gemella* | 0.45 | 2.06 | 1.41 |  |
| *Carnobacterium* |  |  |  | 0.37 |
| *Enterococcus* | 1.43 | 6.32 |  | 0.44 |
| *Blautia* | 2.16 | 0.07 |  | 0.17 |
| *Coprococcus* |  | 0.01 |  |  |
| Lachnospiraceae Incertae Sedis | 0.64 | 0.02 |  | 1.42 |
| Lachnospiraceae uncultured | 1.90 | 0.02 |  |  |
| Peptostreptococcaceae Incertae Sedis |  | 0.01 |  |  |
| Peptostreptococcaceae uncultured |  | 0.12 |  |  |
| *Veillonella* |  | 0.17 |  | 0.48 |
| Erysipelotrichaceae Incertae Sedis | 1.98 | 0.24 |  | 0.20 |
| *Caulobacter* | 0.05 |  |  |  |
| Phyllobacteriaceae uncultured |  |  |  | 0.08 |
| *Rhizobium* | 1.91 | 0.26 |  | 0.98 |
| *Achromobacter* | 0.98 | 0.08 |  |  |
| *Granulicatella* |  |  | 1.21 |  |
| *Bosea* |  |  |  | 0.09 |
| *Ochrobactrum* |  |  | 0.75 | 0.22 |
| *Devosia* |  |  | 1.91 | 1.46 |
| *Sphingomonas* |  |  | 2.71 | 0.33 |
| *Derxia* |  |  |  | 0.51 |
| *Ralstonia* | 1.03 | 0.36 | 0.17 | 1.13 |
| *Acidovorax* | 0.88 | 0.01 | 0.01 | 0.83 |
| *Curvibacter* |  |  |  | 0.09 |
| *Limnohabitans* | 1.07 | 0.17 | 3.01 | 2.35 |
| Neisseriaceae uncultured | 0.12 |  |  |  |
| *Pseudorhodoferax* |  |  | 0.11 | 0.08 |
| Comamonadaceae uncultured |  |  | 0.02 | 0.14 |
| *Undibacterium* |  | 0.13 | 0.50 | 0.40 |
| Methylophilaceae uncultured |  |  |  | 0.10 |
| *Kingella* |  |  |  | 1.52 |
| *Neisseria* |  |  |  | 0.12 |
| *Bdellovibrio* |  |  |  | 1.33 |
| 0319-6G20 uncultured |  |  |  | 0.41 |
| *Sulfurimonas* |  |  |  | 0.41 |
| *Citrobacter* | 0.27 | 0.01 | 0.07 | 0.13 |
| *Escherichia-Shigella* | 0.11 | 0.08 |  |  |
| *Pantoea* | 0.16 |  |  |  |
| *Legionella* |  |  |  | 0.29 |
| *Aggregatibacter* |  |  |  | 0.11 |
| *Haemophilus* |  |  |  | 0.13 |
| *Acinetobacter* |  | 0.01 |  | 0.12 |

|  | **M9-W1** | **M9-W3** | **M9-W6** | **M9-W12** |
| --- | --- | --- | --- | --- |
| **CORE** | **67.65** | **66.54** | **90.42** | **70.87** |
| *Actinomyces* |  |  | 0.06 |  |
| *Gardnerella* |  |  | 0.01 |  |
| *Collinsella* |  |  | 0.02 |  |
| *Rhodococcus* |  |  | 0.01 | 0.18 |
| *Leifsonia* |  |  | 0.17 | 0.27 |
| *Yaniella* |  |  | 0.13 |  |
| *Microbacterium* |  | 0.16 |  | 0.37 |
| *Rothia* |  |  |  | 0.48 |
| *Propionibacterium* | 0.05 | 1.29 |  | 0.90 |
| *Bacteroides* | 4.42 | 0.01 |  |  |
| *Prevotella* |  |  |  | 0.15 |
| *Chryseobacterium* |  |  |  | 1.19 |
| *Ferruginibacter* |  |  |  | 0.24 |
| *Chitinophagaceae* uncultured |  |  |  | 0.92 |
| *Pedobacter* | 0.79 | 10.29 |  | 0.28 |
| Candidate division OD1 uncultured | 0.13 | 0.12 | 1.11 | 4.08 |
| Candidate division SR1 uncultured |  |  |  | 0.50 |
| Candidate division TM7 uncultured |  |  |  | 0.01 |
| Chloroflexi uncultured |  | 1.03 |  |  |
| *Anoxybacillus* |  |  |  | 0.02 |
| *Gemella* | 1.21 |  |  | 0.63 |
| *Enterococcus* |  | 0.10 | 0.01 | 0.71 |
| *Clostridium* |  | 0.04 |  |  |
| *Blautia* |  |  |  | 1.14 |
| Lachnospiraceae Incertae Sedis | 0.02 | 0.07 |  | 1.81 |
| Lachnospiraceae uncultured |  |  |  | 0.12 |
| *Subdoligranulum* |  |  | 0.01 |  |
| *Rhodopseudomonas* |  |  | 0.05 |  |
| *Selenomonas* |  |  |  | 0.17 |
| *Veillonella* |  |  |  | 2.77 |
| Erysipelotrichaceae Incertae Sedis |  |  |  | 0.83 |
| *Ochrobactrum* | 0.01 |  | 0.01 | 0.40 |
| *Devosia* | 0.01 | 0.37 |  |  |
| *Methylobacterium* | 0.01 | 0.53 |  |  |
| *Albibacter* | 0.01 |  |  |  |
| *Aminobacter* | 0.01 |  | 0.10 |  |
| *Phyllobacterium* |  | 0.47 | 0.39 | 1.49 |
| Phyllobacteriaceae uncultured |  |  | 0.41 |  |
| *Rhizobium* |  | 2.43 | 0.96 | 3.69 |
| *Paracoccus* |  |  | 0.42 |  |
| Rhodospirillaceae uncultured |  |  |  | 0.13 |
| *Sphingomonas* |  | 1.16 | 0.59 | 1.37 |
| *Achromobacter* |  | 1.42 | 3.62 | 0.74 |
| *Ralstonia* |  | 1.35 | 0.06 | 1.38 |
| *Acidovorax* | 0.02 | 0.04 |  | 0.10 |
| *Undibacterium* | 0.02 |  |  |  |
| *Limnohabitans* |  | 1.47 | 0.03 | 0.55 |
| *Pelomonas* |  |  |  | 0.48 |
| *Pseudorhodoferax* |  | 3.26 |  | 0.02 |
| Comamonadaceae uncultured |  | 0.17 | 0.01 | 0.08 |
| *Massilia* |  | 1.00 |  |  |
| *Undibacterium* |  | 0.82 | 0.18 |  |
| *Bdellovibrio* |  | 0.98 | 0.52 |  |
| *Citrobacter* | 0.01 | 0.33 | 0.09 | 0.19 |
| *Erwinia* |  | 0.04 | 0.01 |  |
| *Escherichia-Shigella* | 0.02 | 3.29 | 0.45 | 0.01 |
| *Serratia* |  |  | 0.01 |  |
| *Haemophilus* | 24.13 | 0.01 |  |  |
| *Acinetobacter* |  |  |  | 0.35 |

|  | **M10-W1** | **M10-W3** |
| --- | --- | --- |
| **Core** | **91.15** | **63.79** |
| *Gardnerella* | 0.01 |  |
| *Leifsonia* | 0.11 |  |
| *Corynebacterium* |  | 10.27 |
| *Brevibacterium* |  | 0.52 |
| *Agrococcus* |  | 0.01 |
| *Alpinimonas* |  | 0.01 |
| *Rhodoluna* |  | 0.01 |
| *Leucobacter* |  | 0.31 |
| *Microbacterium* | 0.18 | 0.21 |
| *Marmoricola* | 0.09 |  |
| *Propionibacterium* |  | 1.03 |
| *Fluviicola* |  | 0.44 |
| *Bergeyella* |  | 0.06 |
| *Hydrotalea* |  | 0.41 |
| *Pedobacter* | 1.09 | 0.89 |
| Candidate division OD1 uncultured | 0.20 | 0.24 |
| Candidate division TM7 uncultured |  | 0.09 |
| Chloroflexi uncultured |  | 0.01 |
| Cyanobacteria uncultured |  | 0.01 |
| *Gemella* |  | 2.74 |
| *Enterococcus* | 0.38 | 0.77 |
| *Lactococcus* |  | 0.43 |
| Lachnospiraceae Incertae Sedis | 0.02 | 0.05 |
| Lachnospiraceae uncultured |  | 0.13 |
| *Veillonella* | 2.64 | 1.20 |
| *Phyllobacterium* |  |  |
| *Rhizobium* | 0.51 |  |
| Erythrobacteraceae uncultured | 0.05 |  |
| Erysipelotrichaceae Incertae Sedis |  | 0.38 |
| *Devosia* |  | 0.01 |
| *Achromobacter* | 0.04 | 0.17 |
| *Burkholderia* | 0.01 |  |
| *Alcaligenes* |  | 0.24 |
| *Ralstonia* | 0.54 | 0.46 |
| *Acidovorax* | 0.35 | 0.17 |
| *Curvibacter* |  | 0.01 |
| *Limnohabitans* | 0.01 | 0.13 |
| Comamonadaceae uncultured | 0.01 |  |
| *Undibacterium* | 0.21 | 0.40 |
| *Bdellovibrio* |  | 0.01 |
| Aeromonadaceae uncultured |  | 4.37 |
| *Marinobacter* |  | 0.44 |
| *Pseudoalteromonas* |  | 2.64 |
| *Citrobacter* | 0.10 | 0.09 |
| *Haemophilus* | 0.09 |  |
| *Escherichia-Shigella* |  | 0.66 |
| *Halomonas* |  | 2.23 |

**Supplementary Table S2: Relative abundance (%) of the core microbiota at genus level and remaining taxa for each infant, M1 – M10, at week 1, 3, 6 and 12.**

* = Breast milk sample not collected.

|  | **F1-W1** | **F1-W3** | **F1-W6** | **F1-W12** |
| --- | --- | --- | --- | --- |
| *Actinobaculum* | 0.00 | 0.00 | 0.00 | 0.01 |
| *Actinomyces* | 0.00 | 0.00 | 0.00 | 0.73 |
| *Bifidobacterium* | 2.08 | 46.90 | 35.75 | 6.85 |
| *Gardnerella* | 18.04 | 5.85 | 7.48 | 9.82 |
| *Eggerthella* | 0.00 | 0.00 | 0.00 | 0.01 |
| *Corynebacterium* | 0.01 | 0.00 | 0.00 | 0.00 |
| *Rhodococcus* | 0.03 | 0.00 | 0.00 | 0.00 |
| *Rothia* | 0.01 | 0.04 | 0.05 | 0.00 |
| *Propionibacterium* | 0.00 | 0.00 | 0.00 | 0.00 |
| *Bacteroides* | 5.47 | 0.01 | 0.01 | 0.01 |
| *Parabacteroides* | 10.75 | 0.27 | 0.41 | 17.10 |
| *Prevotella* | 0.00 | 0.00 | 0.01 | 0.00 |
| *Gemella* | 0.06 | 0.00 | 0.01 | 0.02 |
| *Staphylococcus* | 41.82 | 15.52 | 10.78 | 0.04 |
| *Enterococcus* | 0.42 | 0.71 | 1.11 | 2.60 |
| *Lactobacillus* | 0.25 | 3.95 | 5.80 | 1.30 |
| *Lactococcus* | 0.02 | 0.00 | 0.00 | 0.00 |
| *Streptococcus* | 10.78 | 11.81 | 36.80 | 0.50 |
| Lachnospiraceae Incertae Sedis | 10.02 | 0.29 | 0.41 | 22.97 |
| Peptostreptococcaceae Incertae Sedis | 0.00 | 0.01 | 0.00 | 0.00 |
| *Veillonella* | 0.00 | 0.02 | 0.01 | 10.33 |
| Erysipelotrichaceae Incertae Sedis | 0.01 | 0.00 | 0.00 | 0.00 |
| *Citrobacter* | 0.12 | 0.00 | 0.00 | 0.08 |
| *Enterobacter* | 0.05 | 0.02 | 0.00 | 0.72 |
| *Erwinia* | 0.00 | 0.01 | 0.00 | 0.03 |
| *Escherichia-Shigella* | 0.04 | 14.56 | 0.62 | 26.81 |
| *Haemophilus* | 0.00 | 0.00 | 0.74 | 0.02 |

|  | **F2-W1** | **F2-W3** | **F2-W6** | **F2-W12** |
| --- | --- | --- | --- | --- |
| *Actinomyces* | 0.00 | 0.02 | 0.02 | 0.03 |
| *Bifidobacterium* | 24.51 | 46.39 | 20.34 | 19.79 |
| *Gardnerella* | 0.00 | 0.01 | 0.01 | 0.01 |
| *Atopobium* | 0.00 | 0.01 | 0.00 | 0.01 |
| *Corynebacterium* | 0.00 | 0.03 | 0.01 | 0.00 |
| *Rhodococcus* | 0.01 | 0.04 | 0.21 | 0.10 |
| *Rothia* | 0.03 | 0.02 | 0.01 | 0.04 |
| *Bacteroides* | 0.00 | 0.01 | 0.01 | 0.06 |
| *Candidate division TM7 uncultured* | 0.00 | 0.00 | 0.00 | 0.11 |
| *Gemella* | 0.09 | 0.01 | 0.01 | 0.07 |
| *Staphylococcus* | 8.87 | 0.52 | 0.01 | 0.02 |
| *Dolosigranulum* | 0.00 | 0.02 | 0.01 | 0.01 |
| *Enterococcus* | 0.17 | 0.27 | 2.15 | 10.62 |
| *Lactobacillus* | 1.38 | 0.84 | 0.01 | 0.01 |
| *Streptococcus* | 2.58 | 5.84 | 0.27 | 3.75 |
| *Clostridium* | 0.00 | 11.02 | 1.67 | 2.13 |
| Peptostreptococcaceae Incertae Sedis | 0.00 | 1.48 | 1.30 | 0.04 |
| *Flavonifractor* | 0.00 | 0.00 | 0.00 | 1.21 |
| *Veillonella* | 5.87 | 8.60 | 12.52 | 14.84 |
| Erysipelotrichaceae Incertae Sedis | 0.00 | 0.00 | 0.00 | 1.30 |
| *Citrobacter* | 43.40 | 10.82 | 28.51 | 4.92 |
| *Enterobacter* | 12.37 | 13.51 | 32.33 | 6.59 |
| *Erwinia* | 0.15 | 0.11 | 0.27 | 1.03 |
| *Escherichia-Shigella* | 0.01 | 0.01 | 0.27 | 29.45 |
| *Haemophilus* | 0.51 | 0.37 | 0.04 | 0.06 |

|  | **F3-W1** | **F3-W3** | **F3-W6** | **F3-W12*** |
| --- | --- | --- | --- | --- |
| *Bifidobacterium* | 0.02 | 23.07 | 8.59 |  |
| *Gardnerella* | 0.01 | 0.00 | 0.02 |  |
| *Atopobium* | 0.00 | 0.00 | 0.04 |  |
| *Eggerthella* | 0.00 | 0.00 | 0.11 |  |
| *Gordonibacter* | 0.00 | 0.00 | 0.00 |  |
| *Corynebacterium* | 0.00 | 0.04 | 0.00 |  |
| *Rothia* | 0.06 | 0.16 | 0.07 |  |
| *Bacteroides* | 0.00 | 0.01 | 44.88 |  |
| *Prevotella* | 0.00 | 0.00 | 0.02 |  |
| *Gemella* | 0.01 | 0.01 | 0.07 |  |
| *Staphylococcus* | 41.47 | 6.65 | 0.35 |  |
| *Dolosigranulum* | 0.00 | 0.01 | 0.00 |  |
| *Enterococcus* | 3.31 | 0.06 | 0.01 |  |
| *Lactobacillus* | 0.00 | 20.50 | 11.18 |  |
| *Streptococcus* | 21.78 | 18.53 | 6.36 |  |
| *Clostridium* | 5.17 | 14.50 | 0.00 |  |
| *Sarcina* | 0.62 | 0.04 | 0.00 |  |
| Lachnospiraceae Incertae Sedis | 0.00 | 0.00 | 5.23 |  |
| *Veillonella* | 2.13 | 4.30 | 19.91 |  |
| Erysipelotrichaceae Incertae Sedis | 25.34 | 11.80 | 3.07 |  |
| *Enterobacter* | 0.00 | 0.01 | 0.02 |  |
| *Escherichia-Shigella* | 0.01 | 0.01 | 0.03 |  |
| *Haemophilus* | 0.05 | 0.26 | 0.01 |  |

|  | **F4-W1** | **F4-W3** | **F4-W6** | **F4-W12** |
| --- | --- | --- | --- | --- |
| *Actinobaculum* | 0.00 | 0.00 | 0.23 | 0.03 |
| *Actinomyces* | 0.00 | 0.46 | 2.15 | 2.76 |
| *Bifidobacterium* | 9.53 | 17.18 | 7.16 | 20.99 |
| *Gardnerella* | 5.86 | 20.26 | 4.76 | 0.96 |
| *Scardovia* | 0.00 | 0.00 | 0.07 | 0.04 |
| *Atopobium* | 0.00 | 0.00 | 0.12 | 0.01 |
| *Collinsella* | 0.59 | 0.18 | 1.41 | 5.02 |
| *Eggerthella* | 0.00 | 0.15 | 1.40 | 0.15 |
| *Corynebacterium* | 0.00 | 0.00 | 0.00 | 0.01 |
| *Rothia* | 0.01 | 0.00 | 0.00 | 0.01 |
| *Propionibacterium* | 0.00 | 0.00 | 0.26 | 0.09 |
| *Bacteroides* | 0.00 | 0.01 | 0.01 | 0.01 |
| *Porphyromonas* | 0.00 | 0.04 | 0.00 | 0.00 |
| *Prevotella* | 0.00 | 0.08 | 0.00 | 0.00 |
| *Gemella* | 0.02 | 0.01 | 0.00 | 0.00 |
| *Staphylococcus* | 1.55 | 0.23 | 0.06 | 0.08 |
| *Dolosigranulum* | 0.00 | 0.00 | 0.01 | 0.00 |
| *Enterococcus* | 1.06 | 0.38 | 3.43 | 3.66 |
| *Lactobacillus* | 0.02 | 1.33 | 10.02 | 7.55 |
| *Streptococcus* | 0.85 | 1.02 | 1.82 | 0.22 |
| *Clostridium* | 0.00 | 0.00 | 0.00 | 1.93 |
| *Anaerococcus* | 0.00 | 0.02 | 0.01 | 0.61 |
| *Finegoldia* | 0.01 | 0.02 | 0.02 | 0.61 |
| *Peptoniphilus* | 0.00 | 0.00 | 0.00 | 0.25 |
| *Coprococcus* | 0.00 | 0.00 | 0.00 | 0.54 |
| Peptostreptococcaceae Incertae Sedis | 0.00 | 0.00 | 0.00 | 0.44 |
| *Negativicoccus* | 0.00 | 0.01 | 0.00 | 0.00 |
| *Veillonella* | 42.81 | 37.09 | 37.73 | 21.04 |
| Erysipelotrichaceae Incertae Sedis | 0.00 | 0.00 | 0.00 | 0.01 |
| *Citrobacter* | 0.00 | 0.00 | 0.02 | 0.00 |
| *Enterobacter* | 0.00 | 0.01 | 0.07 | 0.00 |
| *Erwinia* | 0.00 | 0.00 | 0.01 | 0.00 |
| *Escherichia-Shigella* | 37.66 | 21.47 | 29.22 | 32.73 |
| *Haemophilus* | 0.00 | 0.00 | 0.01 | 0.00 |

|  | **F5-W1** | **F5-W3** | **F5-W6** | **F5-W12** |
| --- | --- | --- | --- | --- |
| *Actinomyces* | 0.43 | 0.00 | 0.00 | 0.07 |
| *Bifidobacterium* | 9.42 | 5.34 | 6.97 | 11.22 |
| *Gardnerella* | 0.01 | 10.29 | 24.23 | 27.60 |
| *Scardovia* | 0.00 | 0.00 | 0.00 | 0.03 |
| *Atopobium* | 0.00 | 0.00 | 0.08 | 0.04 |
| *Collinsella* | 0.17 | 0.00 | 0.00 | 0.00 |
| *Eggerthella* | 0.11 | 0.00 | 0.00 | 0.00 |
| *Corynebacterium* | 0.00 | 0.02 | 0.15 | 0.02 |
| *Rhodococcus* | 0.00 | 0.00 | 0.00 | 0.01 |
| *Rothia* | 0.01 | 0.00 | 0.23 | 0.05 |
| *Bacteroides* | 8.15 | 0.00 | 0.01 | 0.01 |
| *Parabacteroides* | 4.69 | 0.00 | 0.00 | 0.00 |
| *Prevotella* | 0.00 | 0.00 | 0.00 | 0.04 |
| *Gemella* | 0.18 | 0.09 | 0.18 | 0.06 |
| *Staphylococcus* | 11.48 | 2.73 | 6.47 | 0.08 |
| *Granulicatella* | 0.08 | 0.01 | 0.10 | 0.00 |
| *Enterococcus* | 1.58 | 0.00 | 0.01 | 1.40 |
| *Lactobacillus* | 0.01 | 0.02 | 0.00 | 0.00 |
| *Streptococcus* | 22.37 | 24.10 | 30.78 | 13.40 |
| *Peptoniphilus* | 0.00 | 0.00 | 0.00 | 0.01 |
| Lachnospiraceae Incertae Sedis | 2.48 | 0.00 | 0.00 | 0.01 |
| *Veillonella* | 3.37 | 15.07 | 6.82 | 1.67 |
| Erysipelotrichaceae Incertae Sedis | 0.00 | 0.00 | 0.01 | 0.01 |
| *Enterobacter* | 0.00 | 0.00 | 0.01 | 0.02 |
| *Erwinia* | 0.00 | 0.01 | 0.01 | 0.00 |
| *Escherichia-Shigella* | 35.20 | 42.17 | 23.71 | 44.23 |
| *Haemophilus* | 0.23 | 0.12 | 0.21 | 0.01 |

|  | **F6-W1** | **F6-W3** | **F6-W6** | **F6-W12** |
| --- | --- | --- | --- | --- |
| *Actinomyces* | 0.00 | 0.00 | 0.00 | 0.01 |
| *Bifidobacterium* | 0.13 | 0.08 | 0.26 | 0.21 |
| *Gardnerella* | 27.91 | 29.77 | 48.29 | 35.20 |
| *Eggerthella* | 0.00 | 0.00 | 0.00 | 0.29 |
| *Corynebacterium* | 0.00 | 0.00 | 0.00 | 0.01 |
| *Rhodococcus* | 0.00 | 0.00 | 0.00 | 0.73 |
| *Rothia* | 0.03 | 0.05 | 0.04 | 0.00 |
| *Propionibacterium* | 0.00 | 0.00 | 0.04 | 0.07 |
| *Bacteroides* | 0.01 | 0.01 | 0.00 | 0.00 |
| *Elizabethkingia* | 0.00 | 0.00 | 0.01 | 0.00 |
| *Gemella* | 0.18 | 0.17 | 0.10 | 0.00 |
| *Staphylococcus* | 7.49 | 21.27 | 4.62 | 0.09 |
| *Enterococcus* | 0.01 | 0.01 | 6.94 | 1.29 |
| *Lactobacillus* | 59.81 | 45.31 | 30.06 | 4.44 |
| *Streptococcus* | 0.97 | 0.38 | 2.18 | 0.22 |
| *Clostridium* | 0.00 | 0.00 | 0.05 | 0.29 |
| Peptostreptococcaceae Incertae Sedis | 0.02 | 0.00 | 0.01 | 0.44 |
| *Veillonella* | 0.20 | 0.01 | 0.03 | 1.39 |
| Erysipelotrichaceae Incertae Sedis | 0.01 | 0.00 | 0.00 | 4.05 |
| *Citrobacter* | 0.00 | 0.00 | 4.85 | 1.06 |
| *Enterobacter* | 0.00 | 0.00 | 2.21 | 49.07 |
| *Erwinia* | 0.00 | 0.00 | 0.03 | 1.04 |
| *Escherichia-Shigella* | 0.02 | 0.02 | 0.05 | 0.06 |
| *Haemophilus* | 3.20 | 2.92 | 0.23 | 0.03 |

|  | **F7-W1** | **F7-W3** | **F7-W6** | **F7-W12** |
| --- | --- | --- | --- | --- |
| *Actinomyces* | 0.00 | 0.20 | 0.00 | 0.06 |
| *Bifidobacterium* | 41.74 | 34.67 | 32.28 | 43.47 |
| *Gardnerella* | 8.97 | 12.16 | 4.05 | 0.21 |
| *Atopobium* | 0.00 | 0.00 | 0.01 | 0.03 |
| *Corynebacterium* | 0.01 | 0.06 | 0.01 | 0.01 |
| *Rothia* | 0.00 | 1.40 | 0.00 | 0.00 |
| *Bacteroides* | 0.02 | 0.01 | 0.01 | 0.00 |
| *Elizabethkingia* | 0.00 | 0.00 | 0.01 | 0.00 |
| *Gemella* | 0.01 | 0.01 | 0.00 | 0.02 |
| *Staphylococcus* | 32.83 | 15.31 | 0.63 | 0.69 |
| *Dolosigranulum* | 0.01 | 0.06 | 0.01 | 0.01 |
| *Enterococcus* | 0.02 | 0.00 | 0.01 | 5.30 |
| *Lactobacillus* | 0.03 | 0.01 | 0.12 | 1.46 |
| *Streptococcus* | 16.19 | 17.11 | 14.46 | 28.56 |
| *Clostridium* | 0.01 | 16.51 | 0.00 | 0.45 |
| Peptostreptococcaceae Incertae Sedis | 0.00 | 0.00 | 0.00 | 0.18 |
| *Veillonella* | 0.02 | 0.01 | 48.08 | 1.68 |
| Erysipelotrichaceae Incertae Sedis | 0.01 | 0.00 | 0.00 | 0.00 |
| *Enterobacter* | 0.00 | 0.01 | 0.01 | 0.00 |
| *Escherichia-Shigella* | 0.03 | 2.00 | 0.19 | 17.77 |
| *Haemophilus* | 0.03 | 0.01 | 0.00 | 0.00 |

|  | **F8-W1** | **F8-W3** | **F8-W6** | **F8-W12** |
| --- | --- | --- | --- | --- |
| *Actinomyces* | 0.00 | 0.00 | 0.00 | 0.02 |
| *Bifidobacterium* | 7.72 | 2.24 | 2.62 | 8.01 |
| *Gardnerella* | 40.72 | 14.37 | 5.45 | 8.88 |
| *Eggerthella* | 0.00 | 0.00 | 0.00 | 0.14 |
| *Corynebacterium* | 0.01 | 0.00 | 0.00 | 0.00 |
| *Rhodococcus* | 0.00 | 0.00 | 0.00 | 0.01 |
| *Rothia* | 0.01 | 0.01 | 0.03 | 0.01 |
| *Propionibacterium* | 0.00 | 0.00 | 0.00 | 0.02 |
| *Bacteroides* | 0.03 | 0.07 | 16.04 | 43.01 |
| *Elizabethkingia* | 0.00 | 0.00 | 0.00 | 0.01 |
| *Gemella* | 0.06 | 0.02 | 0.00 | 0.01 |
| *Staphylococcus* | 30.43 | 0.34 | 0.00 | 0.10 |
| *Dolosigranulum* | 0.00 | 0.01 | 0.00 | 0.00 |
| *Enterococcus* | 0.00 | 0.08 | 0.51 | 10.83 |
| *Lactobacillus* | 0.01 | 0.01 | 0.15 | 2.60 |
| *Streptococcus* | 14.71 | 0.36 | 0.15 | 0.24 |
| *Clostridium* | 0.00 | 0.00 | 0.15 | 0.01 |
| *Coprococcus* | 0.00 | 0.00 | 0.03 | 0.00 |
| Lachnospiraceae Incertae Sedis | 0.00 | 0.00 | 0.03 | 0.26 |
| Peptostreptococcaceae Incertae Sedis | 0.00 | 0.00 | 0.00 | 0.88 |
| *Veillonella* | 2.16 | 67.31 | 63.52 | 10.03 |
| *Citrobacter* | 0.00 | 0.01 | 0.00 | 0.03 |
| *Enterobacter* | 0.00 | 0.00 | 0.03 | 0.69 |
| *Erwinia* | 0.00 | 0.00 | 0.00 | 0.06 |
| *Escherichia-Shigella* | 3.89 | 15.13 | 11.28 | 14.13 |
| *Haemophilus* | 0.23 | 0.02 | 0.00 | 0.00 |

|  | **F9-W1** | **F9-W3** | **F9-W6** | **F9-W12** |
| --- | --- | --- | --- | --- |
| *Actinomyces* | 0.42 | 0.05 | 0.17 | 1.10 |
| *Bifidobacterium* | 0.02 | 0.01 | 0.03 | 0.00 |
| *Gardnerella* | 0.00 | 0.01 | 0.00 | 0.00 |
| *Eggerthella* | 0.00 | 0.00 | 0.02 | 0.02 |
| *Corynebacterium* | 0.00 | 0.00 | 0.01 | 0.00 |
| *Rothia* | 0.00 | 0.00 | 0.00 | 0.08 |
| *Propionibacterium* | 0.00 | 0.00 | 0.26 | 0.00 |
| *Bacteroides* | 0.01 | 0.01 | 0.00 | 0.01 |
| *Gemella* | 0.06 | 0.16 | 0.00 | 0.00 |
| *Staphylococcus* | 11.76 | 7.06 | 0.67 | 0.05 |
| *Dolosigranulum* | 0.00 | 0.00 | 0.00 | 0.01 |
| *Enterococcus* | 0.00 | 0.00 | 0.00 | 0.04 |
| *Lactobacillus* | 0.01 | 0.01 | 0.54 | 0.21 |
| *Streptococcus* | 27.51 | 57.78 | 40.35 | 0.98 |
| *Clostridium* | 0.00 | 13.27 | 15.35 | 1.02 |
| *Sarcina* | 0.00 | 0.02 | 0.01 | 0.00 |
| *Anaerococcus* | 0.00 | 0.00 | 0.00 | 0.00 |
| *Finegoldia* | 0.00 | 0.00 | 0.00 | 0.00 |
| *Peptoniphilus* | 0.00 | 0.24 | 0.12 | 0.00 |
| *Coprococcus* | 0.00 | 0.00 | 0.00 | 0.00 |
| Lachnospiraceae Incertae Sedis | 0.00 | 0.00 | 16.78 | 32.09 |
| Peptostreptococcaceae Incertae Sedis | 0.00 | 0.04 | 1.33 | 0.10 |
| *Flavonifractor* | 0.00 | 0.00 | 0.00 | 0.00 |
| Ruminococcaceae Incertae Sedis | 0.00 | 0.00 | 0.00 | 0.00 |
| *Megasphaera* | 0.00 | 0.08 | 1.27 | 0.56 |
| *Negativicoccus* | 0.00 | 2.35 | 0.36 | 0.15 |
| *Phascolarctobacterium* | 0.00 | 0.00 | 0.00 | 0.00 |
| *Veillonella* | 27.18 | 11.26 | 15.92 | 22.80 |
| Erysipelotrichaceae Incertae Sedis | 0.00 | 0.00 | 0.00 | 0.00 |
| *Citrobacter* | 0.00 | 0.00 | 0.00 | 0.20 |
| *Enterobacter* | 0.00 | 0.00 | 0.00 | 0.04 |
| *Erwinia* | 0.00 | 0.00 | 0.00 | 0.00 |
| *Escherichia-Shigella* | 0.02 | 0.02 | 0.01 | 0.01 |
| *Haemophilus* | 32.79 | 7.32 | 6.54 | 0.03 |
| *Akkermansia* | 0.00 | 0.00 | 0.00 | 39.49 |

|  | **F10-W1** | **F10-W3** | **F10-W6*** | **F10-W12*** |
| --- | --- | --- | --- | --- |
| *Bifidobacterium* | 1.62 | 6.14 |  |  |
| *Gardnerella* | 0.00 | 0.02 |  |  |
| *Eggerthella* | 0.00 | 0.02 |  |  |
| *Corynebacterium* | 0.01 | 0.09 |  |  |
| *Rhodococcus* | 0.00 | 0.01 |  |  |
| *Rothia* | 0.01 | 0.01 |  |  |
| *Bacteroides* | 0.01 | 0.01 |  |  |
| *Elizabethkingia* | 0.02 | 0.00 |  |  |
| *Flavobacterium* | 0.01 | 0.00 |  |  |
| *Gemella* | 0.01 | 0.00 |  |  |
| *Staphylococcus* | 1.38 | 3.06 |  |  |
| *Dolosigranulum* | 0.00 | 0.01 |  |  |
| *Enterococcus* | 0.48 | 0.01 |  |  |
| *Lactobacillus* | 0.00 | 0.01 |  |  |
| *Streptococcus* | 0.73 | 1.60 |  |  |
| *Clostridium* | 0.47 | 0.01 |  |  |
| Lachnospiraceae Incertae Sedis | 0.01 | 6.76 |  |  |
| *Veillonella* | 52.83 | 3.24 |  |  |
| Erysipelotrichaceae Incertae Sedis | 0.01 | 1.42 |  |  |
| *Enterobacter* | 0.00 | 0.01 |  |  |
| *Escherichia-Shigella* | 41.18 | 77.36 |  |  |
| *Haemophilus* | 1.15 | 0.14 |  |  |

**
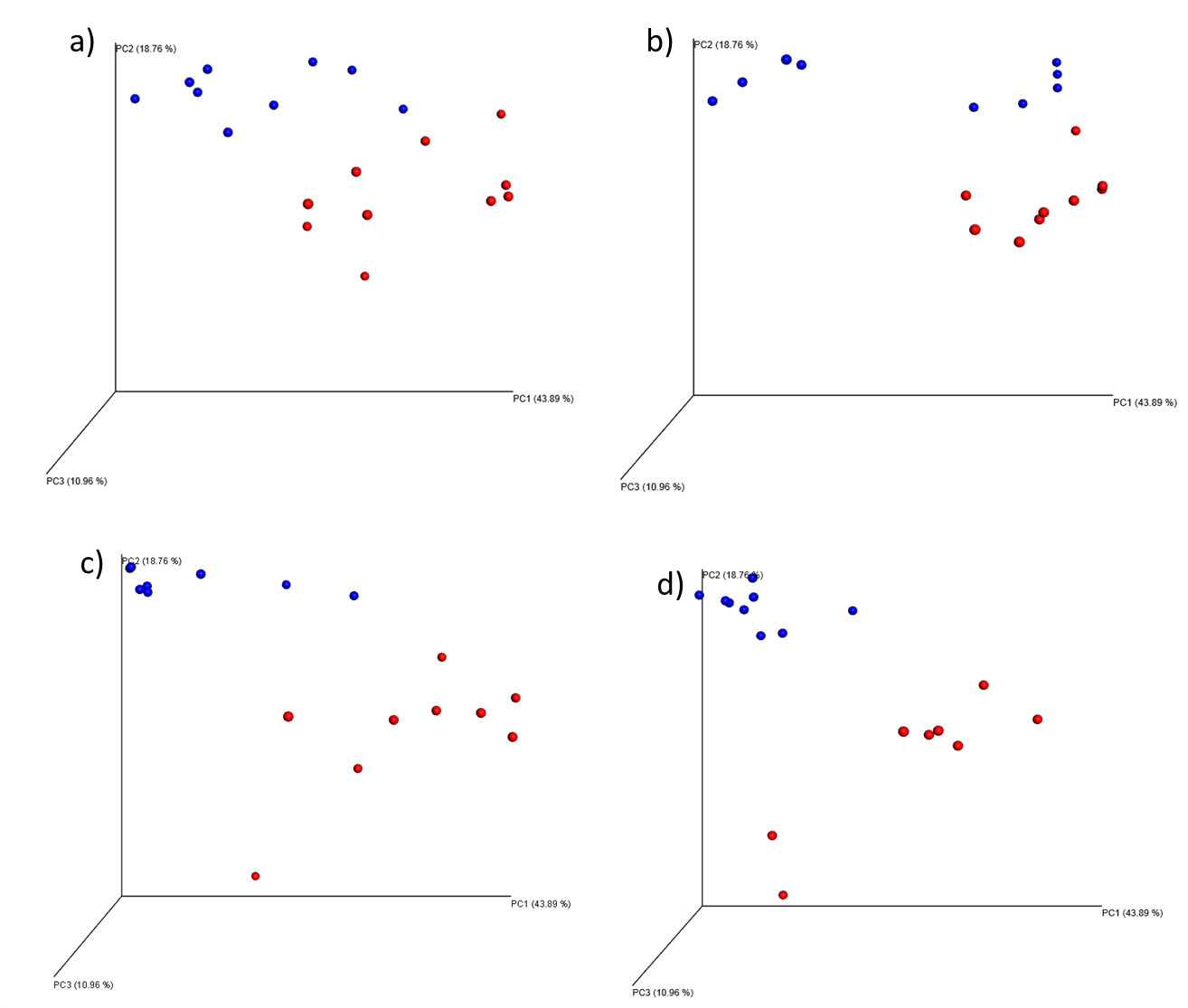
**

**Supplementary Figure S1:**

**Principal coordinates analysis of Weighted UniFrac values between milk (blue) and faecal (red) samples over time with the axes scaled by the percentage of the variance that they contain. a) Week 1, b) Week 3, c) Week 6 and d) Week 12.**

**
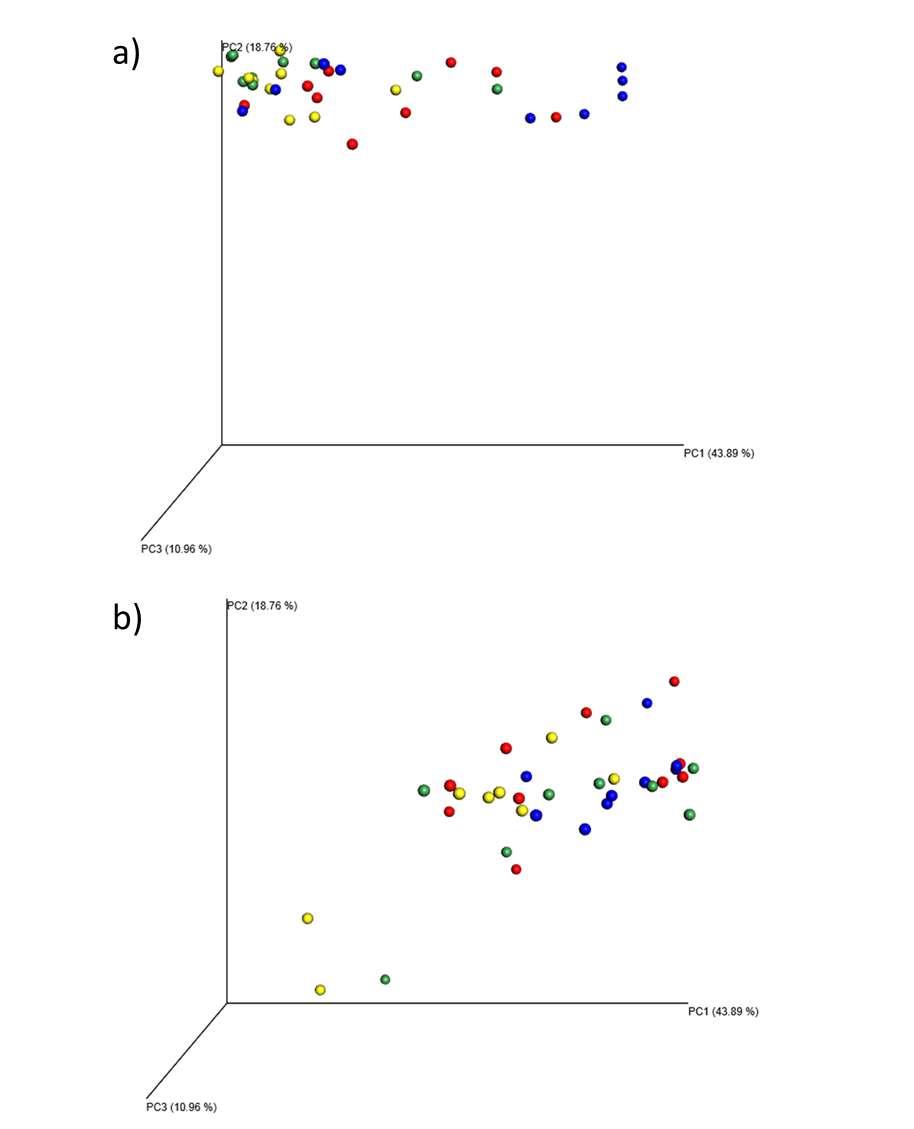
**

**Supplementary Figure S2:**

**Principal coordinates analysis of Weighted UniFrac values between a) milk samples over time and b) faecal samples over time, with the axes scaled by the percentage of the variance that they contain. Red = Week 1, Blue = Week 3, Green = Week 6 and Yellow = Week 12.**

**
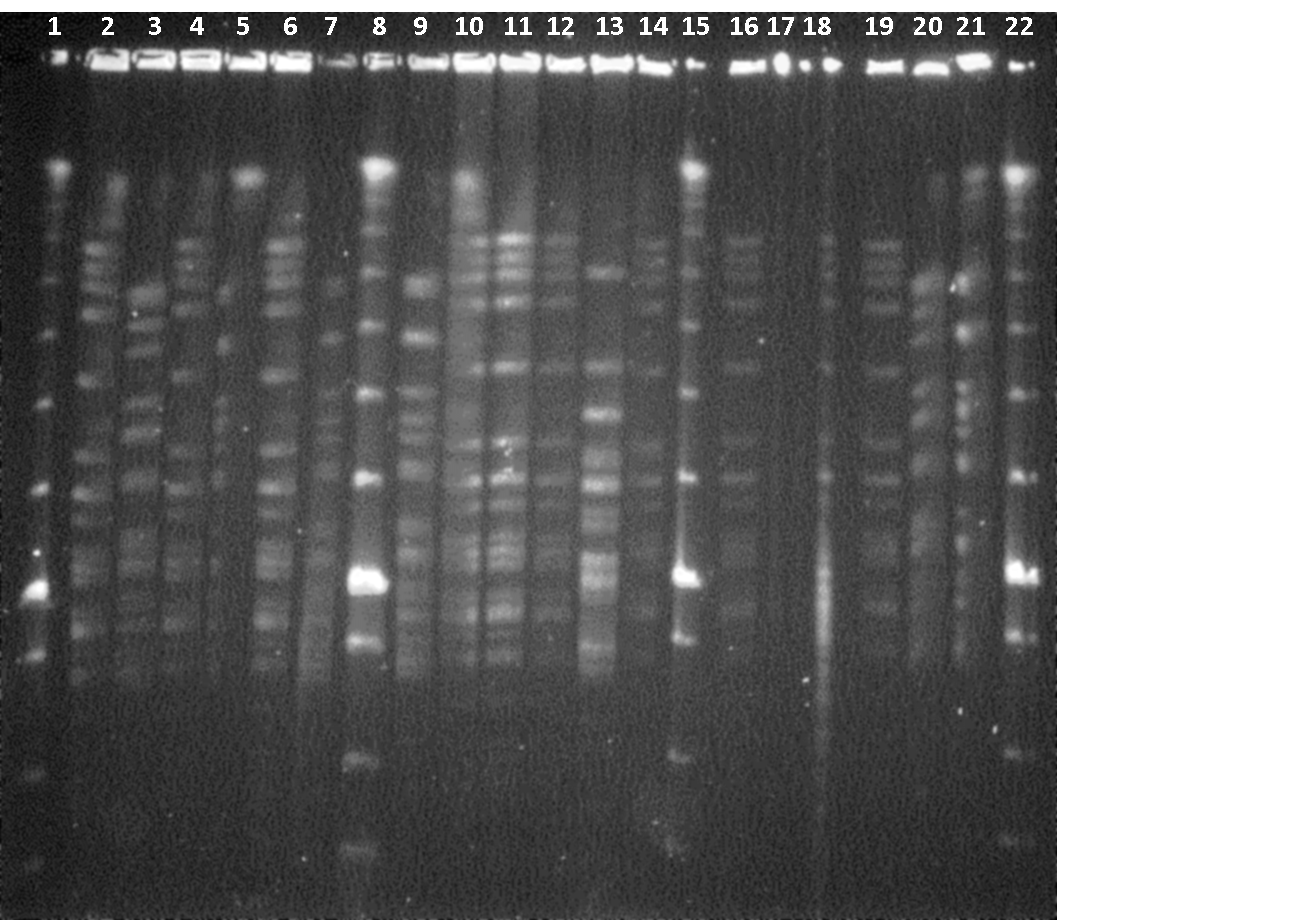
**

**Supplementary Figure S3:**

**Un-edited image of pulse-field gel electrophoresis patterns of XbaI-digested genomic DNA of *B. breve* isolates from human milk and infant faeces. Lanes 1, 8 and 15 contain a low molecular weight marker; Lanes 2, 4, 6 10 and 11 contain isolates from breast-milk; Lanes 12, 14, 16 and 19 contain isolates from infant faeces.**

**
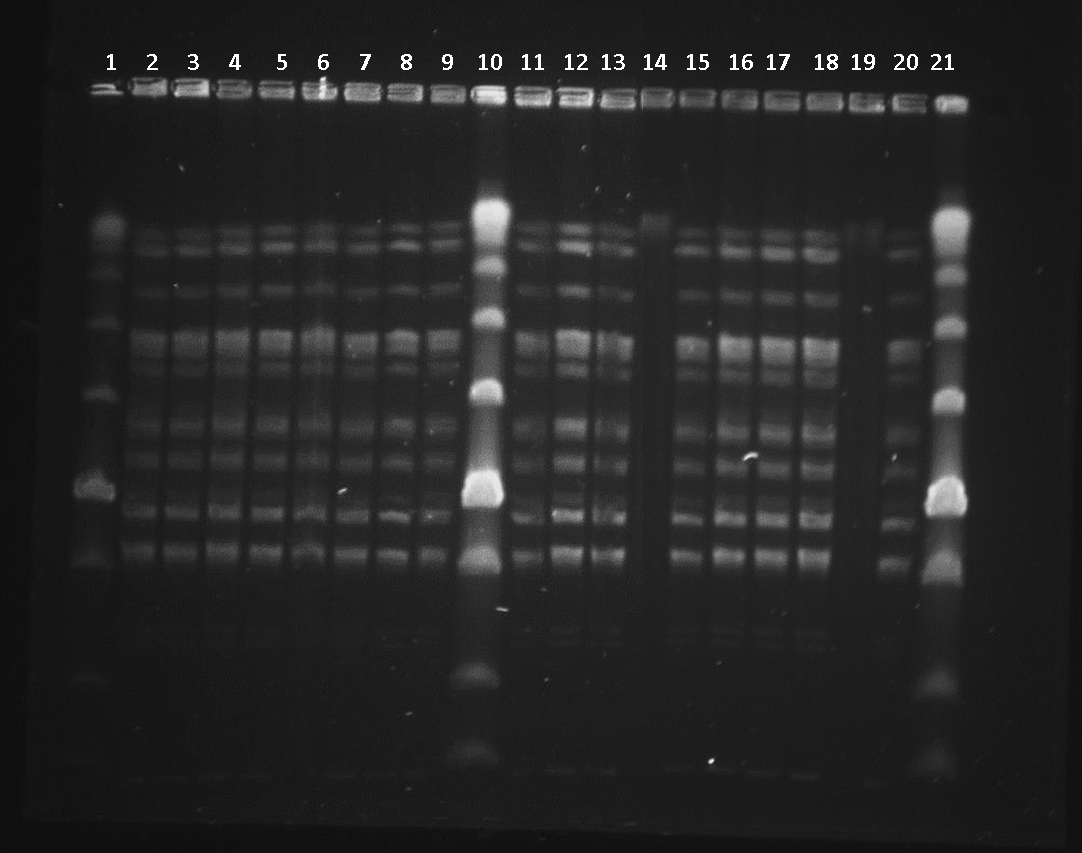
**

**Supplementary Figure S4:**

**Un-edited image of pulse-field gel electrophoresis patterns of ApaI-digested genomic DNA of *L. plantarum* isolates from human milk and infant faeces. Lanes 1, 10 and 21 contain a low molecular weight marker; Lanes 2-9 contain isolates from breast-milk; Lanes 11-20 contain isolates from infant faeces.**
